# Genome-wide identification and characterization of protein phosphatase 2C (PP2C) gene family in sunflower (*Helianthus annuus* L.) and their expression profiles in response to multiple abiotic stresses

**DOI:** 10.1371/journal.pone.0298543

**Published:** 2024-03-20

**Authors:** Nasrin Akter, Md Shohel Ul Islam, Md. Shahedur Rahman, Fatema Tuz Zohra, Shaikh Mizanur Rahman, M. Manirujjaman, Md. Abdur Rauf Sarkar

**Affiliations:** 1 Department of Genetic Engineering and Biotechnology, Faculty of Biological Science and Technology, Jashore University of Science and Technology, Jashore, Bangladesh; 2 Department of Genetic Engineering and Biotechnology, Faculty of Biological Sciences, University of Rajshahi, Rajshahi, Bangladesh; 3 Department of Structural and Cellular Biology, Tulane University School of Medicine, New Orleans, Louisiana, LA, United States of America; University of Delhi, INDIA

## Abstract

Plant protein phosphatase 2C (PP2C) plays vital roles in responding to various stresses, stimulating growth factors, phytohormones, and metabolic activities in many important plant species. However, the PP2C gene family has not been investigated in the economically valuable plant species sunflower (*Helianthus annuus* L.). This study used comprehensive bioinformatics tools to identify and characterize the PP2C gene family members in the sunflower genome (*H*. *annuus* r1.2). Additionally, we analyzed the expression profiles of these genes using RNA-seq data under four different stress conditions in both leaf and root tissues. A total of 121 PP2C genes were identified in the sunflower genome distributed unevenly across the 17 chromosomes, all containing the Type-2C phosphatase domain. HanPP2C genes are divided into 15 subgroups (A-L) based on phylogenetic tree analysis. Analyses of conserved domains, gene structures, and motifs revealed higher structural and functional similarities within various subgroups. Gene duplication and collinearity analysis showed that among the 53 HanPP2C gene pairs, 48 demonstrated segmental duplications under strong purifying selection pressure, with only five gene pairs showing tandem duplications. The abundant segmental duplication was observed compared to tandem duplication, which was the major factor underlying the dispersion of the PP2C gene family in sunflowers. Most HanPP2C proteins were localized in the nucleus, cytoplasm, and chloroplast. Among the 121 HanPP2C genes, we identified 71 miRNAs targeting 86 HanPP2C genes involved in plant developmental processes and response to abiotic stresses. By analyzing cis-elements, we identified 63 cis-regulatory elements in the promoter regions of HanPP2C genes associated with light responsiveness, tissue-specificity, phytohormone, and stress responses. Based on RNA-seq data from two sunflower tissues (leaf and root), 47 HanPP2C genes exhibited varying expression levels in leaf tissue, while 49 HanPP2C genes showed differential expression patterns in root tissue across all stress conditions. Transcriptome profiling revealed that nine HanPP2C genes (HanPP2C12, HanPP2C36, HanPP2C38, HanPP2C47, HanPP2C48, HanPP2C53, HanPP2C54, HanPP2C59, and HanPP2C73) exhibited higher expression in leaf tissue, and five HanPP2C genes (HanPP2C13, HanPP2C47, HanPP2C48, HanPP2C54, and HanPP2C95) showed enhanced expression in root tissue in response to the four stress treatments, compared to the control conditions. These results suggest that these HanPP2C genes may be potential candidates for conferring tolerance to multiple stresses and further detailed characterization to elucidate their functions. From these candidates, 3D structures were predicted for six HanPP2C proteins (HanPP2C47, HanPP2C48, HanPP2C53, HanPP2C54, HanPP2C59, and HanPP2C73), which provided satisfactory models. Our findings provide valuable insights into the PP2C gene family in the sunflower genome, which could play a crucial role in responding to various stresses. This information can be exploited in sunflower breeding programs to develop improved cultivars with increased abiotic stress tolerance.

## 1.0 Introduction

Plants are usually exposed to adverse environmental conditions, including high and low temperatures, salinity, water deficit, heavy metals, herbivory (wounding), and pathogen infection. These conditions have all been demonstrated to modulate harm plant growth and development. Plants have evolved various signaling pathways in response to these stresses and transfer stimuli to cellular compartments [1]. Biochemical and physiological reactions in plants are regulated by reversible protein phosphorylation, a crucial protein modification process that plays an essential function in stress signaling, catalyzed by protein phosphatases (PPs) and protein kinases (PKs) [2]. Protein kinases (PKs) have been thoroughly studied and demonstrated as regulatory factors in response to the diversity of biotic and abiotic stresses [3].

Protein phosphatases (PPs) are categorized into two major classes: protein serine (Ser)/threonine (Thr) phosphatases (PSPs) and protein tyrosine phosphatases (PTPs) [4]. Protein phosphatase 1 (PP1) and protein phosphatase 2 (PP2) are two subcategories of PSPs according to biochemical properties using activators and inhibitors. Based on cofactor requirements, PP2 proteins are further classified into protein phosphatases 2A (PP2As), protein phosphatases 2B (PP2B), and protein phosphatases 2C (PP2C). Whereas PP2As have no metal ion requirements for activity, PP2Bs, require a calcium ion (Ca^2+^) and a magnesium (Mg^2+^). PP2Cs, on the other hand, require a calcium ion (Ca^2+^) and a manganese (Mn^2+^) ion [5]. Moreover, according to amino acid sequences, PSPs can be categorized into two subclasses: phosphoprotein metallophosphatase (PPM) containing Mg^2+^ or Mn^2+^ ions, which includes the PP2C group, and phospho-protein phosphatase (PPP), which includes PP1, PP2A, and PP2B groups [6].

PP2C genes are evolutionarily conserved from prokaryotes to eukaryotes and found in animals, plants, fungi, bacteria, and archaea. They modulate stress-signaling mechanisms by reversing stress-induced protein kinase (PK) cascades [7]. PP2Cs are the largest phosphatase gene family (60–65% of all phosphorylases) in plants, with a unique structure containing a conserved catalytic domain at the N-terminus or C-terminus and an un-conserved domain at the opposite region [8]. PP2C genes have various functions in signal transduction pathways for their structural diversity [9]. A total of 80, 62, 78, 131, and 257 PP2C genes have been identified using various bioinformatics techniques in Arabidopsis (*Arabidopsis thaliana*) [10], strawberry (*Fragaria vesca*) [11], rice (*Oryza sativa*) [12], mustard (*Brassica rapa*) [13], and wheat (*Triticum aestivum*) [14], respectively. According to the evolutionary relationships, the PP2C gene family in Arabidopsis was classified into ten subgroups (A-J) [15]. Subgroup A includes nine members, 6 of them (ABI1, ABI2, AHG1, HAB1, HAB2, and AHG3/ATPP2CA) negatively modulate ABA signaling and others three (HAI1, HAI2/AIP1, and HAI3) responded individually to stress [16]. Two candidate inhibitors of ABI1 protein phosphatase were identified, and they have the potential to play a role in modulating ABA responses [17]. The PP2C gene family plays crucial roles in the modulation of stress signaling and plant development. In tomatoes, PP2C plays a significant role in fruit maturity by modulating the gene expression of ethylene [18]. However, limited PP2C genes in economically important plant species have been functionally investigated. To our knowledge, no genome-wide identification of the PP2C gene family of H. annuus has been reported. Therefore, it is important to identify and analyse the functions of the PP2C gene family in sunflowers (*Helianthus annuus* L.).

Sunflower (*Helianthus annuus* L.) is an essential annual dicot plant from the Asteraceae family native to North America and cultivated worldwide as an oilseed crop [19]. It is the fourth most economically profitable oilseed plant after soybean, rapeseed, and safflower and is used to obtain edible oil for medical purposes and as an ornamental plant [20]. Approximately 47,347,175 tons of sunflowers are produced yearly (www.atlasbig.com). Sunflower has several agricultural benefits, such as fast growth, limited water requirement, and extended flowering period [21]. The phenolic compounds, flavonoid compounds, polyunsaturated fatty acids, and vitamins of sunflower provide antioxidant, antimicrobial, anti-hypertensive, cardiovascular, anti-inflammatory, and wound-healing benefits [22]. Nonetheless, due to the recent worldwide climate-change scenario, many crop species with a high economic value, including sunflower, have been affected by various stresses that severely hampered the yield and oil quality in these crop species [23–25]. Advanced breeding approaches must be developed to overcome the global climate change behavior and meet food security challenges. Comprehensive bioinformatics analysis of the target HanPP2C gene family members and their validation by the expression level within a short period have become more useful in breeding programs to improve this crop species. Previous studies identified the PP2C genes of various economically essential plant genomes, such as 81 PP2C genes (0.29%) were found among 27,029 protein-coding genes of Arabidopsis [10, 26] genome. Moreover, 78 PP2C genes (0.14%) among 56,221 protein-coding genes, 134 PP2C genes (0.29%) among 46,430 protein-coding genes, and 91 PP2C genes (0.26%) among 34,727 protein-coding genes were identified in the rice [10, 27], soybean [28, 29], and tomato [30, 31] genome, respectively.

However, there are limitations to identify HanPP2C gene family and analyze their expression in terms of human resources, time, well-equipped laboratories, and experimental expenditure. Despite extensive laboratory-based research on target gene family members, we can collect substantial genomic information from a variety of important plant species using various comprehensive bioinformatics tools, which reduce labor-inputs, funding-inputs, and time investments. In this study, we used integrated bioinformatics analysis to find out more information about the sunflower PP2C (HanPP2C) genes, such as genome-wide identification, physical and chemical properties, phylogenetic comparison, genomic evolution, gene structure, conserved domain, motifs, gene duplication, chromosome mapping, subcellular localization, cis-acting regulatory elements, tissue-specific expression analysis under various stress conditions, and 3D homology modeling of selected proteins. The findings presented here will build the core foundation for functional investigations on the HanPP2C genes and offer brilliant opportunities to improve this crop species in future breeding programs.

## 2.0 Materials and methods

### 2.1 Database search and retrieval of PP2C protein sequences in sunflower (*H*. *annuus*) genome

Initially, the *A*. *thaliana* PP2C DNA-binding domains were used to retrieve PP2C gene-encoding proteins in the *H*. *annuus* (*Helianthus annuus* r1.2) genome at Phytozome v13 (https://phytozome-next.jgi.doe.gov/) using BLASTp (Protein-basic local alignment search tool) [32], with an expected (E) threshold value of -1, a comparison matrix (BLOSUM62), and other default parameters. The Pfam PP2C domain “PF00481” was also used as a query term to ensure the presence of PP2C proteins. Furthermore, retrieved amino acid sequences were analyzed for conserved PP2C domains using SMART (Simple Modular Architecture Research Tool, http://smart.embl-heidelberg.de/) [33] and the NCBI CDD (Conserved Domain Database) (https://www.ncbi.nlm.nih.gov/Structure/cdd/wrpsb.cgi) [34] with default parameters. Predicted proteins lacking the PP2C conserved domain (PF00481) were excluded from the candidate list. These genes encoding PP2C domains were renamed according to the order of their physical chromosomal positions.

### 2.2 Determination of physio-chemical properties of sunflower PP2C proteins

The number of amino acid residues, molecular weight, isoelectric value (pI), instability index, aliphatic index, and grand average of hydropathicity (GRAVY) of PP2C proteins were predicted using the ProtParam online tool (http://web.expasy.org/protparam/) [35].

### 2.3 Phylogenetic analysis of Arabidopsis and sunflower PP2Cs

*A*. *thaliana* and *H*. *annuus* PP2C protein sequences were retrieved from Phytozome v13, and a phylogenetic tree was constructed using MEGA11 software [36] with the ClustalW program [37, 38] for sequence alignment (S1 Data). The Maximum Likelihood (ML) method with default parameters was used, except for a 1000 bootstrap value to support branch values and Pearson correction. The constructed tree was then uploaded to iTOL v6.7.4 (https://itol.embl.de/) [39] for appropriate representation.

### 2.4 Gene structure analysis

To determine the gene structure of PP2Cs, CDS and genomic DNA sequences in FASTA format were retrieved from the Phytozome v13 (S2 and S3 Data). Moreover, the "gf3" file of the sunflower genome data was retrieved from Phytozome v13. Gene Structure Display Server (GSDS v2.0) [40] (available at http://gsds.cbi.pku.edu.cn/) was used to analyze the *H*. *annuus* genome.

### 2.5 Conserved domain and motif analysis

The InterPro database (http://www.ebi.ac.uk/interpro/) was used to predict the PP2C conserved domains, and TBtools software-v1.116 [41] was used to display the results. The structural motifs of the PP2C protein sequences were analyzed using the Multiple EM for Motif Elicitation (MEME) (https://meme-suite.org/meme/tools/meme) (http://meme.nbcr.net/meme/) tools of MEME-suite (https://meme-suite.org/meme/) [42], selecting a maximum number of motifs 20 with other default parameters (S4 Data). MEME and the motif scanning method (MSA), enabled by the MEME web interface, were used to visualize the motifs.

### 2.6 Gene duplication analysis and synonymous (Ks) and non-synonymous (Ka) substitution ratios calculation

Synonymous (Ks) and non-synonymous (Ka) substitution ratios of the sunflower PP2C gene family were determined using the Ka/Ks calculation tool (http://services.cbu.uib.no/tools/kaks) with HanPP2C CDS sequences of duplicated genes. The rates of molecular evolution were determined for each pair of paralogous genes using the Ka/Ks ratios. Duplication and time of divergence (million years ago, MYA) (T) of the HanPP2C gene were calculated by T = Ks/2λ (λ = 6.5×10^−9^) [43].

### 2.7 Collinearity and synteny analysis of the PP2C gene family of sunflower

For collinearity and synteny analysis, gene duplications of *H*. *annuus* and *A*. *thaliana* PP2C genes were analyzed, and the identified HanPP2C collinear pairs and their collinear pairs with Arabidopsis were illustrated using TBtools version-v1.116 [41].

### 2.8 Analysis of chromosomal location

The sunflower (*H*. *annuus*) "Hannuus_494_r1.0 gf3" file was retrieved from the Phytozome v13 database. Information on the chromosomal length, start, and end points of 121 HanPP2C gene locations were collected using TBtools software version-v1.116 [41]. The distribution of HanPP2C genes across the chromosomes was mapped using the collected information through the MapGene2Chrom web v2 (MG2C) web server (http://mg2c.iask.in/mg2c_v2.0/) [44].

### 2.9 Prediction of the subcellular localization of PP2c family members of sunflower

The subcellular localization of the PP2C proteins in sunflowers was predicted using the Wolf PSORT (https://wolfpsort.hgc.jp/) online tool [45, 46]. The predicted protein signals of each HanPP2C gene were demonstrated using TBtools version-v1.116 [41].

### 2.10 Cis-acting regulatory elements analysis of HanPP2C gene promoters

To investigate the cis-acting regulatory elements (CAREs), the 2000 bp upstream promoter region of each HanPP2C sequence was obtained from the Phytozome v13 database. Using the plant CARE database (http://bioinformatics.psb.ugent.be/webtools/plantcare/html/), the CAREs of the PP2C genes were predicted [47]. The predicted CAREs were categorized and illustrated as a heatmap using TBtools version-v1.116 [41]

### 2.11 Putative microRNA target site analysis

The micro-RNA (miRNA) datasets of sunflowers were downloaded from the plant microRNA encyclopedia (http://pmiren.com/) [48]. The CDS sequences of all sunflower HanPP2C genes were analyzed for sequences complementary to miRNAs (*Helianthus annuus* (Sunflower), unigene, DFCI Gene Index (HAGI), version 6, released on 2009_05_24) with an expectation value of 5.0 and other default parameters of psRNATarget (https://www.zhaolab.org/psRNATarget/analysis) [49] to identify miRNAs that potentially target the sunflower HanPP2C genes.

### 2.12 Transcriptomic expression pattern analysis in various stress conditions

Previously generated RNA-seq transcriptomic data of an inbred sunflower line (HA412-HO; PI 642777) seedlings (*H*. *annuus*) treated with four abiotic stresses (C = Control, DD = Dry Down, N = Nutrient, P = PEG (Polyethylene glycol), and S = Salt)) in root and leaf tissues, maintained for 20 days after germination to examine the specific expression pattern of PP2C genes were utilized [50]. Treatments were implemented at the V2 stage of sunflower seedling development [51]. Dry-down treatments were performed by repeatedly drying the top-down soil. Sunflower seedlings were surface-watered daily using deionized (DI) water only to induce low-nutrient stress. PEG (polyethylene glycol) at 8.25% by volume and -0.25 MPa [52] osmotic challenge was induced for PEG treatment. In contrast, 100 mM NaCl solution was used for daily watering to investigate salt treatment. Fragments Per Kilobases Per Million Mapping Reads (FPKM) values from RNA-seq data were log2 transformed for expression profiling. Using TBtools version-v1.116 [39], a heatmap was generated to illustrate the expression patterns via hierarchical clustering.

### 2.13 Homology-based modeling of HanPP2C proteins

The 3D (three-dimensional) homology-based models of sunflower PP2C proteins were predicted using YASARA homology software (version 22.9.24.W.64) [53]. Homology modeling was performed with PSI-BLAST iterations and a PSI-BLAST E-value set to 3 and 0.1, respectively. The alignments per template parameter were set to 5, and the terminal extension and loop number per sample were set to 10 and 50, respectively.

## 3.0 Results

### 3.1 Identification of PP2C genes in sunflower genome

This study identified 121 genes encoded PP2C proteins in the sunflower (*H*. *annuus* r1.2) genome at Phytozome v13 through BLASTp using Arabidopsis PP2C genes as references. The hidden Markov (HMM) model was used to ensure the presence of the protein phosphatase 2C (PP2C) domain using the SMART and Pfam databases. The identified PP2C genes in the sunflower genome were labeled as HanPP2C1 to HanPP2C121 based on their chromosomal distribution and respective order. The basic information such as gene ID, the amino acid (aa) length, molecular weight (MW), isoelectric point (pI), instability index, and hydropathicity of 121 HanPP2C genes were analyzed (Table 1). The length of proteins varied from 121 aa residues (HanPP2C12) to 1070 aa residues (HanPP2C109), most of which were between 300 aa and 400 aa. MW and pI values ranged from 13.24 kDa (HanPP2C12) to 119.34 kDa (HanPP2C70) and 4.72 (HanPP2C15) to 9.50 (HanPP2C113), respectively. Out of 121 HanPP2C, 77 HanPP2C genes (63.64%) showed an unstable instability index, whereas 44 HanPP2C genes showed a stable instability index. According to the hydropathicity (GRAVY) result, all HanPP2C genes except HanPP2C12, HanPP2C22, and HanPP2C84 were hydrophilic.

**Table 1:** List of 121 HanPP2C genes and their basic physio-chemical characterization.

SI	Gene identifier	Gene name	Size (aa)	Mass (kDa)	pI	Instability index	Aliphatic index	GRAVY
1	HanXRQChr01g0009741	HanPP2C1	375	41.89698	8.86	37.38	89.12	-0.342
2	HanXRQChr01g0011751	HanPP2C2	379	41.11442	6.83	56.33	74.56	-0.329
3	HanXRQChr01g0011811	HanPP2C3	413	44.66328	5.98	33.46	91.11	-0.143
4	HanXRQChr01g0025621	HanPP2C4	359	39.34535	5.37	45.58	84.82	-0.234
5	HanXRQChr01g0025691	HanPP2C5	366	39.94415	5.29	43.59	86.42	-0.170
6	HanXRQChr01g0025731	HanPP2C6	368	40.31644	5.38	43.49	85.41	-0.251
7	HanXRQChr01g0025741	HanPP2C7	370	40.53978	5.46	40.84	83.11	-0.204
8	HanXRQChr01g0027041	HanPP2C8	375	42.28834	8.92	51.25	89.12	-0.320
9	HanXRQChr02g0032441	HanPP2C9	445	49.29850	5.23	44.76	77.30	-0.452
10	HanXRQChr02g0034061	HanPP2C10	361	39.62926	4.93	34.27	66.73	-0.583
11	HanXRQChr02g0037691	HanPP2C11	430	46.57690	5.30	39.86	84.98	-0.253
12	HanXRQChr02g0050381	HanPP2C12	121	13.24012	4.96	37.27	95.95	0.176
13	HanXRQChr02g0051821	HanPP2C13	398	44.26348	7.65	35.41	87.19	-0.274
14	HanXRQChr03g0063961	HanPP2C14	368	41.21855	5.06	43.31	85.30	-0.310
15	HanXRQChr03g0065811	HanPP2C15	361	39.19640	4.72	40.94	78.09	-0.339
16	HanXRQChr03g0068321	HanPP2C16	290	31.61499	6.84	37.40	88.79	-0.298
17	HanXRQChr03g0068701	HanPP2C17	369	41.29740	9.41	39.35	92.11	-0.347
18	HanXRQChr03g0078771	HanPP2C18	332	36.78569	5.72	36.66	85.75	-0.261
19	HanXRQChr03g0080041	HanPP2C19	326	36.17882	5.14	35.62	72.98	-0.394
20	HanXRQChr03g0080761	HanPP2C20	370	41.07351	4.93	40.95	89.81	-0.216
21	HanXRQChr03g0083811	HanPP2C21	366	40.09840	5.35	33.48	82.27	-0.201
22	HanXRQChr04g0094871	HanPP2C22	691	76.62216	6.50	37.40	91.95	0.013
23	HanXRQChr04g0112331	HanPP2C23	339	37.20215	5.47	41.73	86.64	-0.188
24	HanXRQChr04g0123011	HanPP2C24	469	51.81909	5.68	44.26	77.29	-0.482
25	HanXRQChr05g0134881	HanPP2C25	679	76.23651	5.44	39.52	80.37	-0.487
26	HanXRQChr05g0135271	HanPP2C26	381	41.41462	5.91	45.69	73.15	-0.401
27	HanXRQChr05g0141351	HanPP2C27	304	33.61354	8.00	45.29	82.30	-0.262
28	HanXRQChr05g0142971	HanPP2C28	390	43.35236	9.12	44.09	85.21	-0.318
29	HanXRQChr05g0144811	HanPP2C29	382	41.88359	5.35	55.92	81.18	-0.266
30	HanXRQChr05g0148521	HanPP2C30	355	39.03277	7.48	44.21	76.31	-0.193
31	HanXRQChr05g0153691	HanPP2C31	327	35.83314	5.01	40.51	68.62	-0.475
32	HanXRQChr05g0162881	HanPP2C32	277	30.43712	5.86	42.31	83.07	-0.481
33	HanXRQChr06g0177471	HanPP2C33	176	19.42093	5.03	34.46	92.44	-0.222
34	HanXRQChr06g0179071	HanPP2C34	401	43.84654	6.76	41.70	76.08	-0.382
35	HanXRQChr06g0179631	HanPP2C35	434	47.93919	5.80	45.69	79.47	-0.363
36	HanXRQChr06g0182161	HanPP2C36	356	39.51285	5.79	39.69	79.07	-0.257
37	HanXRQChr07g0192431	HanPP2C37	138	15.47332	5.01	47.13	82.75	-0.298
38	HanXRQChr07g0192671	HanPP2C38	407	44.41256	5.00	33.55	75.36	-0.393
39	HanXRQChr07g0201631	HanPP2C39	221	24.20246	7.86	48.81	54.66	-0.416
40	HanXRQChr07g0201641	HanPP2C40	369	40.31969	5.62	55.43	72.60	-0.337
41	HanXRQChr07g0201651	HanPP2C41	355	39.09953	5.47	51.36	86.14	-0.225
42	HanXRQChr07g0203201	HanPP2C42	359	39.69406	6.72	48.60	77.88	-0.384
43	HanXRQChr07g0203271	HanPP2C43	359	39.73104	6.22	50.37	79.78	-0.362
44	HanXRQChr07g0203281	HanPP2C44	359	39.69406	6.72	48.60	77.88	-0.384
45	HanXRQChr07g0206421	HanPP2C45	393	43.92030	4.92	44.72	86.46	-0.283
46	HanXRQChr08g0208311	HanPP2C46	365	40.19079	6.84	35.71	80.33	-0.411
47	HanXRQChr08g0214071	HanPP2C47	370	40.51171	7.46	58.65	72.19	-0.408
48	HanXRQChr08g0216371	HanPP2C48	387	42.35720	4.92	56.24	88.68	-0.223
49	HanXRQChr08g0221041	HanPP2C49	294	33.71974	4.94	37.83	78.81	-0.736
50	HanXRQChr08g0229111	HanPP2C50	348	37.63776	5.95	53.78	81.21	-0.180
51	HanXRQChr08g0234791	HanPP2C51	699	78.49744	5.39	46.01	74.84	-0.606
52	HanXRQChr08g0236301	HanPP2C52	365	39.91518	5.38	51.52	77.84	-0.300
53	HanXRQChr08g0237391	HanPP2C53	422	45.84803	7.46	46.77	78.34	-0.354
54	HanXRQChr09g0238691	HanPP2C54	367	40.12392	4.97	56.18	78.88	-0.320
55	HanXRQChr09g0240611	HanPP2C55	458	50.58546	6.02	42.70	87.71	-0.115
56	HanXRQChr09g0240651	HanPP2C56	401	44.56282	6.50	45.98	88.75	-0.138
57	HanXRQChr09g0240681	HanPP2C57	347	37.67854	5.14	34.28	88.59	-0.078
58	HanXRQChr09g0240711	HanPP2C58	396	43.94396	5.58	40.74	91.87	-0.076
59	HanXRQChr09g0243021	HanPP2C59	363	40.37565	4.92	45.28	84.82	-0.246
60	HanXRQChr09g0246131	HanPP2C60	389	42.77076	6.28	43.78	84.47	-0.271
61	HanXRQChr09g0246191	HanPP2C61	290	31.03829	4.80	40.06	76.03	-0.314
62	HanXRQChr09g0248001	HanPP2C62	477	51.66718	5.01	42.92	84.80	-0.231
63	HanXRQChr09g0250051	HanPP2C63	385	41.70503	5.20	47.59	76.91	-0.301
64	HanXRQChr09g0253021	HanPP2C64	360	39.43252	5.72	47.53	71.28	-0.387
65	HanXRQChr09g0257251	HanPP2C65	386	42.63873	8.56	43.84	95.75	-0.161
66	HanXRQChr09g0259441	HanPP2C66	379	41.41696	5.38	61.68	80.82	-0.392
67	HanXRQChr09g0267901	HanPP2C67	364	38.88429	5.33	38.07	93.16	-0.058
68	HanXRQChr09g0276231	HanPP2C68	240	26.57113	4.91	36.05	83.67	-0.258
69	HanXRQChr09g0276631	HanPP2C69	484	52.60793	5.24	36.73	89.65	-0.139
70	HanXRQChr10g0281961	HanPP2C70	1060	119.34027	6.00	43.14	78.30	-0.416
71	HanXRQChr10g0288231	HanPP2C71	380	42.29755	8.84	35.30	97.47	-0.194
72	HanXRQChr10g0294661	HanPP2C72	284	30.73242	4.99	41.96	83.70	-0.178
73	HanXRQChr10g0295911	HanPP2C73	281	30.82479	7.82	24.45	83.99	-0.458
74	HanXRQChr10g0297851	HanPP2C74	373	41.84086	8.90	41.33	91.69	-0.320
75	HanXRQChr10g0297981	HanPP2C75	389	43.44950	9.15	51.54	87.92	-0.257
76	HanXRQChr10g0298501	HanPP2C76	651	73.02899	5.62	41.67	86.85	-0.263
77	HanXRQChr10g0302681	HanPP2C77	288	31.84118	6.14	32.03	84.27	-0.360
78	HanXRQChr10g0303991	HanPP2C78	432	47.19151	5.17	42.64	83.70	-0.300
79	HanXRQChr10g0312351	HanPP2C79	469	50.34849	5.06	34.67	99.98	-0.002
80	HanXRQChr10g0312771	HanPP2C80	395	42.92967	5.43	58.13	84.30	-0.291
81	HanXRQChr11g0327411	HanPP2C81	702	78.32601	5.18	39.33	78.13	-0.539
82	HanXRQChr11g0335211	HanPP2C82	468	51.03565	5.57	33.33	77.69	-0.414
83	HanXRQChr11g0338121	HanPP2C83	280	30.24791	6.41	24.64	83.64	-0.386
84	HanXRQChr11g0338821	HanPP2C84	377	41.20701	5.16	33.27	102.39	0.026
85	HanXRQChr11g0340081	HanPP2C85	356	39.20432	5.39	39.55	74.78	-0.311
86	HanXRQChr11g0349591	HanPP2C86	381	42.61491	8.68	48.45	88.71	-0.237
87	HanXRQChr11g0353861	HanPP2C87	381	41.82227	6.01	42.33	85.46	-0.227
88	HanXRQChr12g0361131	HanPP2C88	283	31.48001	7.69	28.59	85.09	-0.351
89	HanXRQChr12g0367111	HanPP2C89	387	42.10493	5.23	48.57	86.15	-0.181
90	HanXRQChr13g0390901	HanPP2C90	388	43.42666	7.24	41.54	88.94	-0.295
91	HanXRQChr13g0393121	HanPP2C91	423	46.43670	4.97	44.13	65.74	-0.598
92	HanXRQChr13g0393511	HanPP2C92	369	41.29437	8.64	36.36	92.11	-0.184
93	HanXRQChr13g0400271	HanPP2C93	589	64.77298	5.90	50.96	83.11	-0.306
94	HanXRQChr13g0405491	HanPP2C94	390	42.33734	7.93	59.76	81.69	-0.232
95	HanXRQChr13g0411021	HanPP2C95	322	35.71754	5.48	30.64	77.24	-0.334
96	HanXRQChr13g0411331	HanPP2C96	281	31.14746	5.51	33.79	91.89	-0.240
97	HanXRQChr13g0413411	HanPP2C97	321	35.16463	6.20	48.81	75.08	-0.256
98	HanXRQChr13g0413441	HanPP2C98	355	38.96749	6.67	32.94	85.97	-0.183
99	HanXRQChr13g0414541	HanPP2C99	390	43.36362	8.18	43.69	93.21	-0.206
100	HanXRQChr13g0416691	HanPP2C100	763	84.32233	5.68	48.89	74.97	-0.519
101	HanXRQChr14g0439281	HanPP2C101	375	41.69017	5.91	41.97	91.20	-0.246
102	HanXRQChr14g0459551	HanPP2C102	372	40.65212	6.11	47.69	79.09	-0.288
103	HanXRQChr15g0484771	HanPP2C103	391	43.44855	8.91	46.82	93.50	-0.236
104	HanXRQChr15g0486111	HanPP2C104	343	38.56543	5.98	44.53	89.45	-0.202
105	HanXRQChr15g0492141	HanPP2C105	419	46.64879	5.28	52.25	79.79	-0.365
106	HanXRQChr15g0493161	HanPP2C106	395	43.89697	6.85	55.75	88.48	-0.265
107	HanXRQChr15g0493551	HanPP2C107	383	41.79925	5.08	56.07	74.62	-0.428
108	HanXRQChr15g0494551	HanPP2C108	208	23.23431	5.40	41.32	84.28	-0.368
109	HanXRQChr16g0507681	HanPP2C109	1070	118.07836	4.90	36.82	87.89	-0.191
110	HanXRQChr16g0507761	HanPP2C110	147	16.98257	5.55	42.56	98.84	-0.107
111	HanXRQChr16g0510191	HanPP2C111	373	41.52033	8.76	34.21	80.16	-0.315
112	HanXRQChr16g0512471	HanPP2C112	184	19.95248	5.94	62.53	89.67	-0.212
113	HanXRQChr16g0514801	HanPP2C113	280	30.57056	9.50	36.24	81.18	-0.505
114	HanXRQChr16g0514931	HanPP2C114	373	41.86490	9.19	39.91	91.93	-0.324
115	HanXRQChr16g0529151	HanPP2C115	426	45.82944	7.84	34.78	83.26	-0.153
116	HanXRQChr16g0531691	HanPP2C116	398	43.91015	8.51	46.92	93.87	-0.179
117	HanXRQChr17g0542581	HanPP2C117	458	50.26241	5.03	47.40	69.59	-0.338
118	HanXRQChr17g0545241	HanPP2C118	400	44.66295	7.65	30.31	90.15	-0.285
119	HanXRQChr17g0551771	HanPP2C119	360	39.82523	5.71	48.75	81.69	-0.272
120	HanXRQChr17g0551841	HanPP2C120	425	45.63019	8.60	39.06	82.16	-0.197
121	HanXRQChr01g0009741	HanPP2C121	448	49.41322	5.19	38.95	74.20	-0.465

**Note:** aa: number of amino acids; pI: theoretical isoelectric point; MW: molecular weight (kDa). GRAVY: grand average of hydropathicity (GRAVY< 0, hydrophilic protein/GRAVY>0, hydrophobic protein) [54], Instability Index (<40, the protein is stable/>40, the protein is unstable) [55].

### 3.2 Phylogenetic analysis of Arabidopsis and sunflower PP2Cs

**Fig 1:**
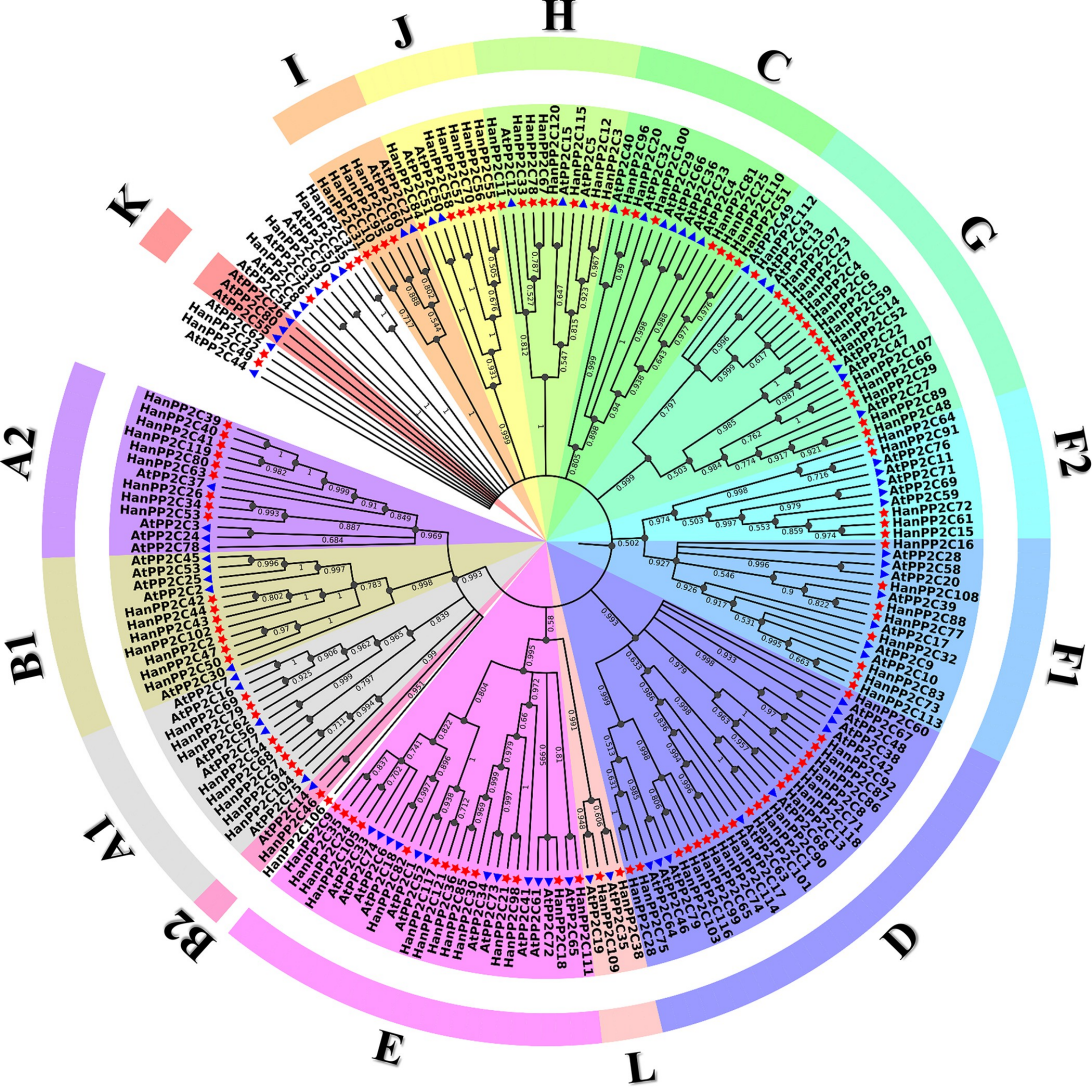
The phylogenetic relationship among 121 HanPP2C and 80 AtPP2C gene families, respectively. Multiple sequence alignments (MSA) were generated using MEGA 11.0 [36] with full-length protein sequences of 121 HanPP2C and 80 AtPP2C members by the Maximum Likelihood (ML) method. Bootstrap values, with 1000 replications for each branch, were calculated. The HanPP2Cs were categorized into 15 subgroups (A1, A2, B1, B2, C, D, E, F1, F2, G, H, I, J, K, and L), each marked by distinct colors. Meanwhile, the AtPP2C genes were marked by a blue triangle, and a red star marked the HanPP2C genes. Additionally, 7 HanPP2Cs were included in the out-group and are represented without color. The corresponding bootstrap values for each branch are displayed within the respective node clusters.

We constructed a phylogenetic tree to determine the relationship between 121 HanPP2C and 80 AtPP2C proteins based on multiple sequence alignment (MSA) (Fig 1). This analysis revealed that each subfamily contained PP2C genes from Arabidopsis and sunflower, and genes of both species tended to form separate branches within each subgroup. As a result, HanPP2C genes from the same sub-clade tended to cluster together, while AtPP2C genes from the same sub-clade also formed clusters. The 121 HanPP2C genes were categorized into 15 subgroups while HanPP2C22, HanPP2C37, HanPP2C45, HanPP2C49, HanPP2C76, HanPP2C93, and HanPP2C106 were not clustered with any subgroup (S5 Data). Based on previous studies, we used the same nomenclature for the 15 common subgroups (A-L) [56]. In this analysis, the genes of groups A, B, and F were divided into subgroups, which are named subgroups A1, A2, B1, B2, F1, and F2. Subgroups A-L (except subgroup K) contain eight, nine, seven, one, seven, twenty, fourteen, eight, five, fifteen, eight, four, six, zero, and two HanPP2C genes, respectively. However, D (20 HanPP2Cs) and G (15 HanPP2C) were relatively larger subgroups, and B2 (1 HanPP2C) and L (2 HanPP2C) were the smallest among the subgroups. The distribution of AtPP2C and HanPP2C genes was comparable except for subgroup K, which contains only AtPP2C genes.

### 3.3 Gene structure analysis

**Fig 2:**
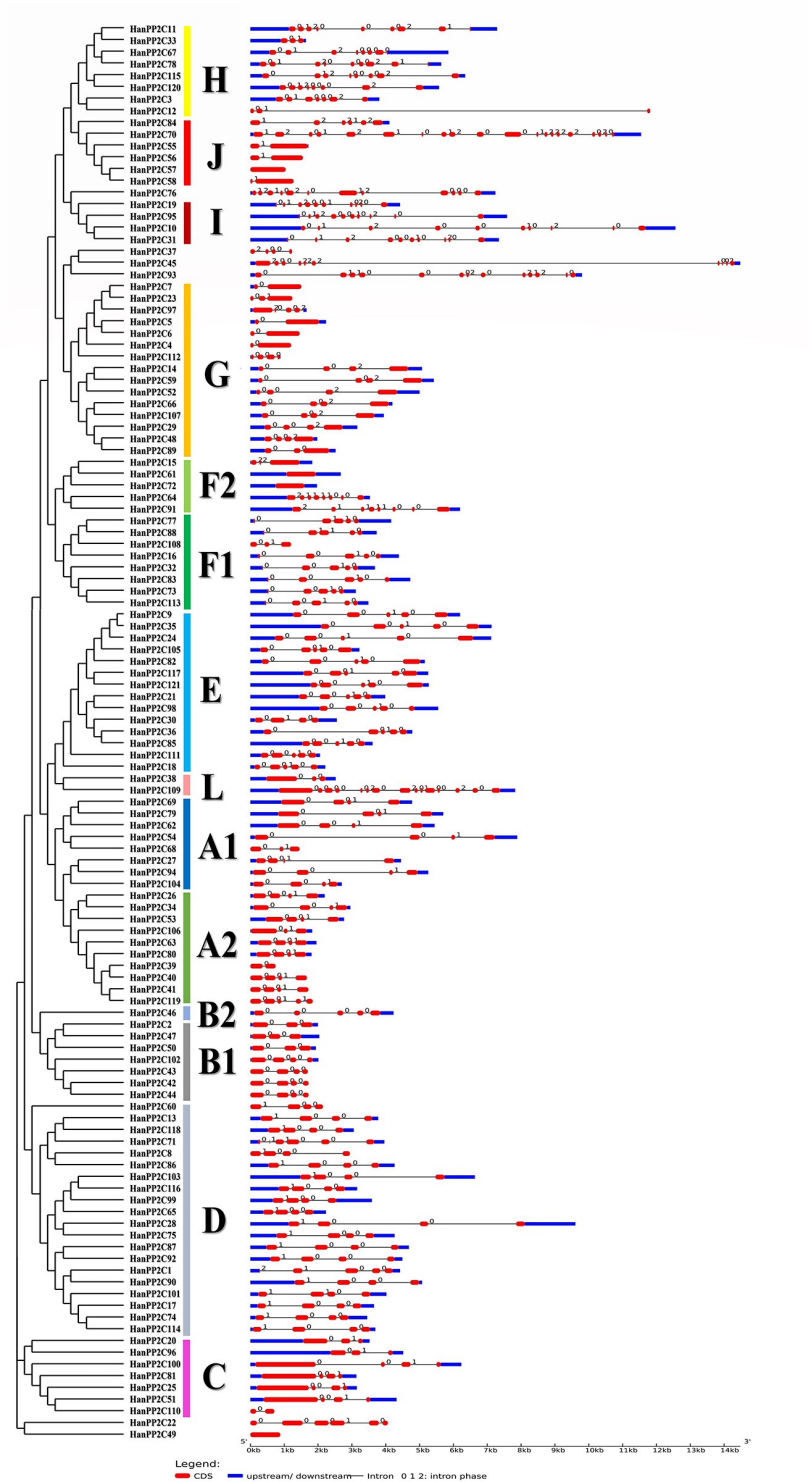
The gene structure of HanPP2C genes. Gene structure analyses for HanPP2C genes were carried out using Gene Structure Display Server (GSDS 2.0, http://gsds.cbi.pku.edu.cn/index.php). The lengths of exons and introns for each HanPP2C gene are demonstrated proportionally. Gene families are categorized and colored based on their phylogenetic relationships. For all HanPP2C genes, black lines represent introns, red-bold lines represent exons, and blue-bold lines represent 5’ and 3’ untranslated regions (UTR). The structure of each HanPP2C gene exon/intron is displayed proportionally according to the scale mentioned at the bottom.

To understand the gene structure of HanPP2C, we analyzed the exon-intron structural patterns based on phylogenetic relationships. This analysis is essential as it is a crucial evolutionary indicator for gene families. The structural patterns of HanPP2C genes demonstrated consistency with phylogenetic analysis (Fig 2 and S6 Data). In subgroup C, HanPP2C96 contains the most extended 5’ and 3’ untranslated region (UTR) region, and HanPP2C4, HanPP2C6, HanPP2C8, HanPP2C12, HanPP2C22, HanPP2C23, HanPP2C37, HanPP2C39, HanPP2C40, HanPP2C41, HanPP2C42, HanPP2C43, HanPP2C44, HanPP2C49, HanPP2C56, HanPP2C57, HanPP2C58, HanPP2C60, HanPP2C68, HanPP2C108, HanPP2C110, and HanPP2C119 have no UTR. The number of introns varied from 0 to 19 among the 121 genes, and only four genes (HanPP2C49, HanPP2C57, HanPP2C61, and HanPP2C72) have no introns whereas HanPP2C70 contains 19 introns. In addition, HanPP2C70 in group J has 20 exons, the highest exon number among all groups, and HanPP2C45 has the largest gene segment of 14 kb long. Furthermore, HanPP2C49 and HanPP2C57 have only one exon and no intron and UTR regions, the lowest exon number among all HanPP2C genes. Investigated gene structures revealed that most of the HanPP2C members of the same group have similar numbers of exon/intron but varied in length. The HanPP2C gene structure was highly similar in each group; however, variations in exon/intron patterns were identified in some genes. This result suggests that the HanPP2C genes were relatively conserved in evolution, preserving the integrity of gene structure and could cause a slight change in functions.

### 3.4 Protein conserved domain analysis of HanPP2C genes

**Fig 3:**
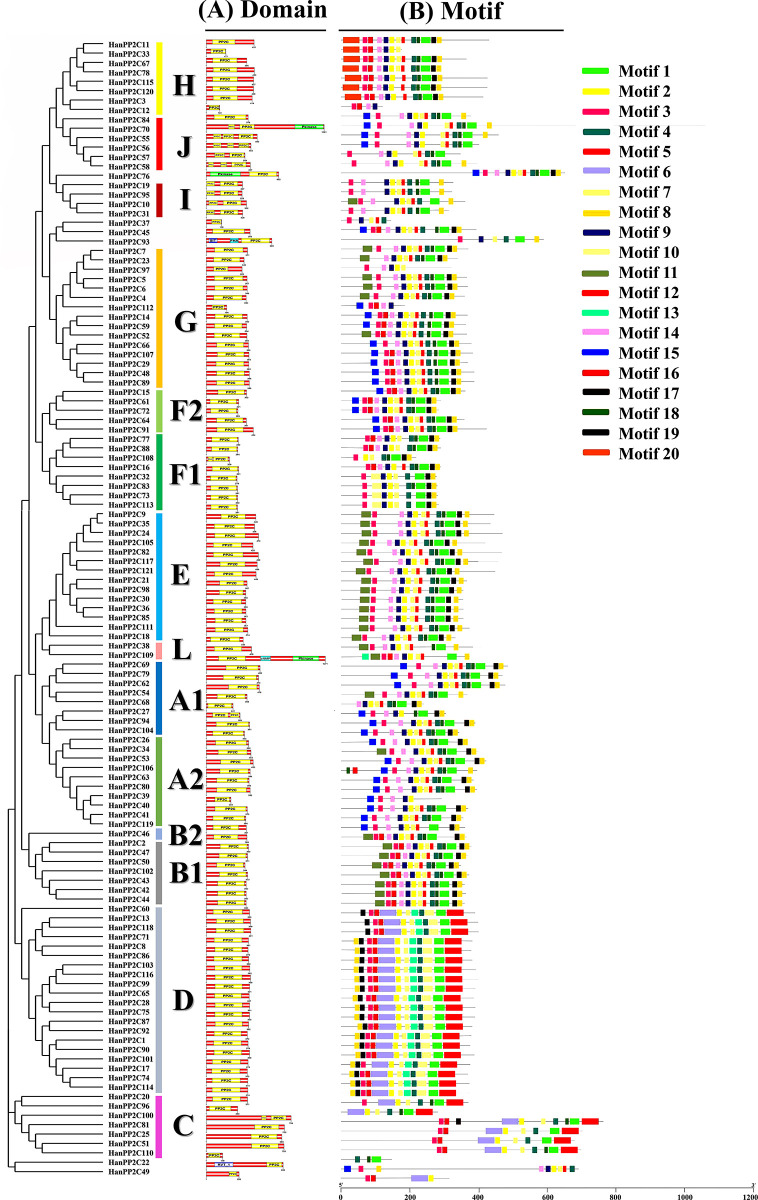
The distribution of conserved domains and motifs in HanPP2C proteins (A-B). (A) The positions of each conserved domain are demonstrated in differently colored boxes, with the domain names presented on the inside of the each domain. (B) The identification of conserved motifs in HanPP2C proteins was carried out using the Multiple EM for Motif Elicitation (MEME) (https://meme-suite.org/meme/tools/meme) tools at MEME-suite (https://meme-suite.org/meme/) [42], with a maximum of 20 motifs selected. Each motif is represented by a specific-colored box aligned on the right side of the figure. Different colors indicate individual motifs identified within each protein domain.

The conserved domain analysis showed that 120 HanPP2C genes (excluding HanPP2C49) contained the typical structural PP2C domain, which is associated with other domains such as PKc-like superfamily, cNMP binding, FHA superfamily, RT-like superfamily, and NADB Rossmann (Fig 3A). Among these, three HanPP2C proteins (HanPP2C70, HanPP2C76, and HanPP2C109) contained PKc-like superfamily domain, whereas HanPP2C109 also exhibited another conserved domain (RT-like superfamily) in addition to the PP2C domain and PKc-like superfamily. Furthermore, HanPP2C22, HanPP2C32, and HanPP2C93 each possessed a single conserved domain (NADB Rossmann, FHA superfamily, cNMP binding domain, respectively) and the typical domain. Consequently, it would be interesting to analyze the diverse biological roles of HanPP2C proteins with these distinctive domains.

### 3.5 Protein motifs analysis

Conserved motifs in HanPP2C proteins were investigated using MEME tools (Fig 3B; S1 Fig and Table 2). The composition of motifs tended to be assembled based on the phylogeny of this study, indicating that the motif compositions within each HanPP2C subgroup were similar but varied among groups. According to this study, motifs 1, 2, 3, and 7 were present in all subgroups, while some were specific to particular subgroups. For instance, motifs 4, 8, and 18 were absent in subgroups L, C, and D but were present in all other subgroups. Motifs 9, 12, and 14 were found in all subgroups except C and D. In contrast, some motifs were unique to only one or two subgroups. For example, motifs 5 and 6 were exclusively present in subgroup C (excluding HanPP2C110) and D subgroups. Motifs 10 and 13 were identified in subgroups F1, D, and L, D, respectively. Motifs 19 and 20 were present only in subgroups D and H (except HanPP2C12), respectively. This investigation suggests that specific motifs may govern the distinct functions of genes within different subgroups. Furthermore, HanPP2C genes within the same subgroups exhibited similar motif distributions, strongly indicating close evolutionary relationships among these genes.

**Table 2:** Conserved motifs in the amino acid sequences of HanPP2C proteins.

Motif	Width	Consensus sequence
1	29	LTPEDEFLILASDGLWDHMSNQEAVDIVR
2	15	LYVANCGDSRAVLCR
3	15	HFFGVYDGHGGPEAA
4	15	GQLAMSRAIGDWYLK
5	50	PRNGIAKRLVKAALQEAAKKREMRYHDLKKIERGVRRHFHDDITVIVIFL
6	50	EQHSMSAEVIKKAFLATEEGFLSIVRQQWPVCPQIAAVGSCCLVGVICNG
7	15	AIQLTTDHKPNREDE
8	15	KRHSCDNITCIVVQF
9	15	CFYSGTTAVTAIIQG
10	29	KPEFNREPLYAKFRLPEPFRRPILSAEPS
11	29	NERYGVYTQQGKRGTMQDAMIVWEDFGCH
12	11	RERIEACGGYV
13	21	ELHSLHPDDPHIVVMKHNVWR
14	15	WKEAMNKAFQKMDKE
15	21	RWGFCSVCGRRRYMEDFYICI
16	15	YVNENLFNNIMKFPQ
17	15	NDPQRAAKQLVKEAL
18	11	PWVIAEPEVTQ
19	15	NMLLEDQSQVESGPL
20	49	HHQTVPLSVLLKRELANEKMERPEIVYGQANQSKKGEDYFLIKTECQRV

### 3.6 Ka/Ks analysis of HanPP2C gene family

**Fig 4:**
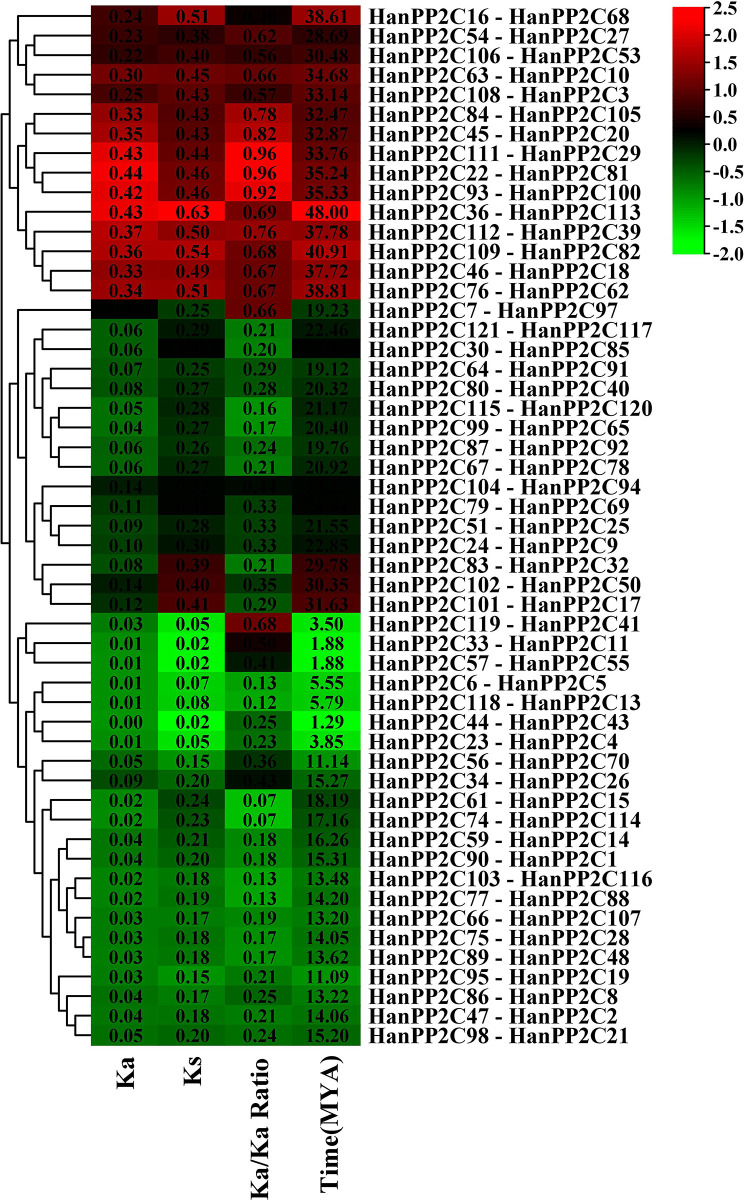
The estimation of gene duplication time for different paralogous gene pairs among sunflower HanPP2C genes, based on Ks and Ka values. Gene duplication analyses were conducted using TBtools software version-v1.116 [41]. Ka presents the number of nonsynonymous substitutions per nonsynonymous site, while Ks represents the number of synonymous substitutions per site. The ratio of nonsynonymous (Ka) to synonymous (Ks) changes is represented by Ka/Ks.

To determine the evolutionary relationships and selection pressures acting on the protein-coding HanPP2C gene, we calculated the Ka (nonsynonymous), Ks (synonymous) values, and Ka/Ks ratios for 53 homologous pairs (Fig 4 and S7 Data). These values are essential in the evolutionary analysis of the HanPP2C gene family members, as they play a pivotal role. If the value of Ka/Ks is less than 1, the duplicated gene pairs may have evolved through purifying selection, also known as negative selection. A Ka/Ks value equal to 1 indicates neutral selection, while a Ka/Ks value greater than 1 shows positive selection. This analysis revealed that the Ka/Ks ratio for 53 duplicated pairs of HanPP2C genes ranged from 0.07 to 0.96 (which is less than 1) observed in the HanPP2C74-HanPP2C114 pair, and HanPP2C111-HanPP2C29 pair, respectively indicating their evolution through purifying selection. Here, the divergence time (T = Ks/2s) among 53 pairs of duplicated HanPP2C genes was also analyzed, using a clock-like rate of 6.5×10^−9^ mutations per synonymous site per year. The results demonstrated that divergence events in HanPP2C genes were estimated to range from 1.29 to 47.10 MYA (Million Years Ago) for the pair HanPP2C44-HanPP2C43 and HanPP2C36-HanPP2C113, respectively. This finding helps to elucidate the extensive evolutionary history of the HanPP2C gene family.

### 3.7 Collinearity and synteny analysis of the PP2C gene family of sunflower

**Fig 5:**
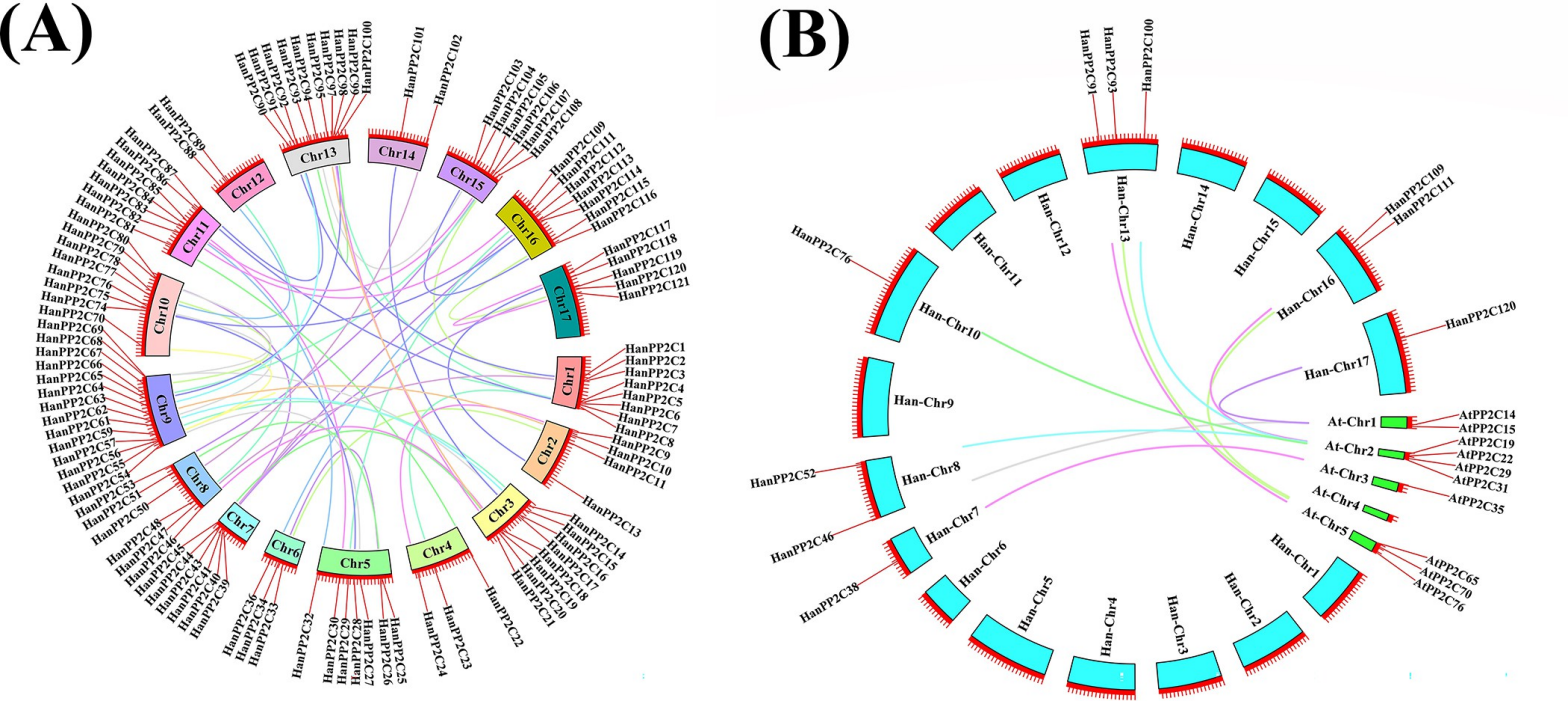
The collinearity analysis of the PP2C gene family in Sunflower and the synteny analysis of PP2Cs between sunflower and Arabidopsis (A and B). (A) Various colored rectangles represent chromosomes 1–17. Different colored lines represent collinear blocks within the sunflower genome, while the colored lines linked between chromosomes represent segmental and tandem duplicated gene pairs, respectively. (B) The aqua-colored blocks represent the collinear blocks in sunflower, while light green-colored blocks represent the synteny blocks in Arabidopsis. Different colored lines represent the syntenic gene pairs of PP2Cs. The aqua rectangles represent sunflower chromosomes (1–17), and the light green rectangles represent Arabidopsis chromosomes (1–5), respectively.

The investigation of collinearity and synteny analysis aimed to determine the distinctions in replication and evolutionary relationships within the HanPP2C gene family. Collinearity, a specific type of synteny, requires the conservation of gene order. The study revealed that 53 gene pairs within the HanPP2C gene family showed a collinear relationship, with chromosome 9 belonging to the highest number of collinear genes (14 pairs), while chromosomes 12 and 14 contain the lowest number, only two pairs each (Fig 5A). Notably, among the 53 pairs of genes, genes of each 40 pairs were found to be located within the same subgroup, indicating strong homology among these pairs. The remaining 13 pairs, however, were distributed across different subgroups. Remarkably, within the collinear pairs HanPP2C20-HanPP2C45 and HanPP2C53-HanPP2C106, the genes HanPP2C45 and HanPP2C106 are located in an outgroup. Furthermore, the conserved motifs and gene structures of genes within the same subfamily were nearly identical. Additionally, synteny analysis identified ten pairs of PP2C genes demonstrating homology in Arabidopsis and sunflower (Fig 5B). Remarkably, the highest number of 3 pairs of sunflower homolog genes is located on chromosome 13, while a minimum of 1 pair of genes was distributed in chromosomes 7, 10, and 17, respectively. These results strongly indicate a high degree of homology between AtPP2C and HanPP2C genes.

### 3.8 Chromosomal distribution and gene duplication events for HanPP2C genes

To visualize the genomic organization of HanPP2C genes, we determined their locations on chromosomes based on information retrieved from the Phytozome v13 genome database. This analysis revealed that 121 HanPP2C genes were unevenly distributed across 17 chromosomes, forming clusters of two or more genes on the same chromosome (S2 Fig). The majority of PP2C genes were located on chromosome 09 (16 genes), followed by chromosomes 10 and 13 (11 genes each), and then Chr07 (9 genes). Chromosomes 01, 03, 05, 08, and 16 each contained eight genes, while the lowest HanPP2C genes were found on Chromosomes 12 and 14 (only two genes). Notably, HanPP2C42 and HanPP2C44 were found to encode similar amino acid sequences but were located on different chromosomal positions within chromosome 07. Generally, no correlation was observed between the number of HanPP2C genes on a chromosome and its length. Furthermore, most genes on the same chromosome did not share a common subclade in the phylogenetic tree, indicating that different HanPP2C genes on a single chromosome can encode proteins with distinct functions. In plant development, genome duplication occurs through two major duplication patterns: segmental and tandem duplication. Tandem duplications refer to closely linked genes found within 200 kb of each other on the same chromosome; otherwise, they were classified as segmental duplications. In this investigation, out of 53 pairs of HanPP2C genes, 48 gene pairs were identified as segmental duplications, denoted by light blue arrows, and five pairs as tandem duplications indicated by light orange lines.

### 3.9 Prediction of subcellular localization of PP2C family members of sunflower

**Fig 6:**
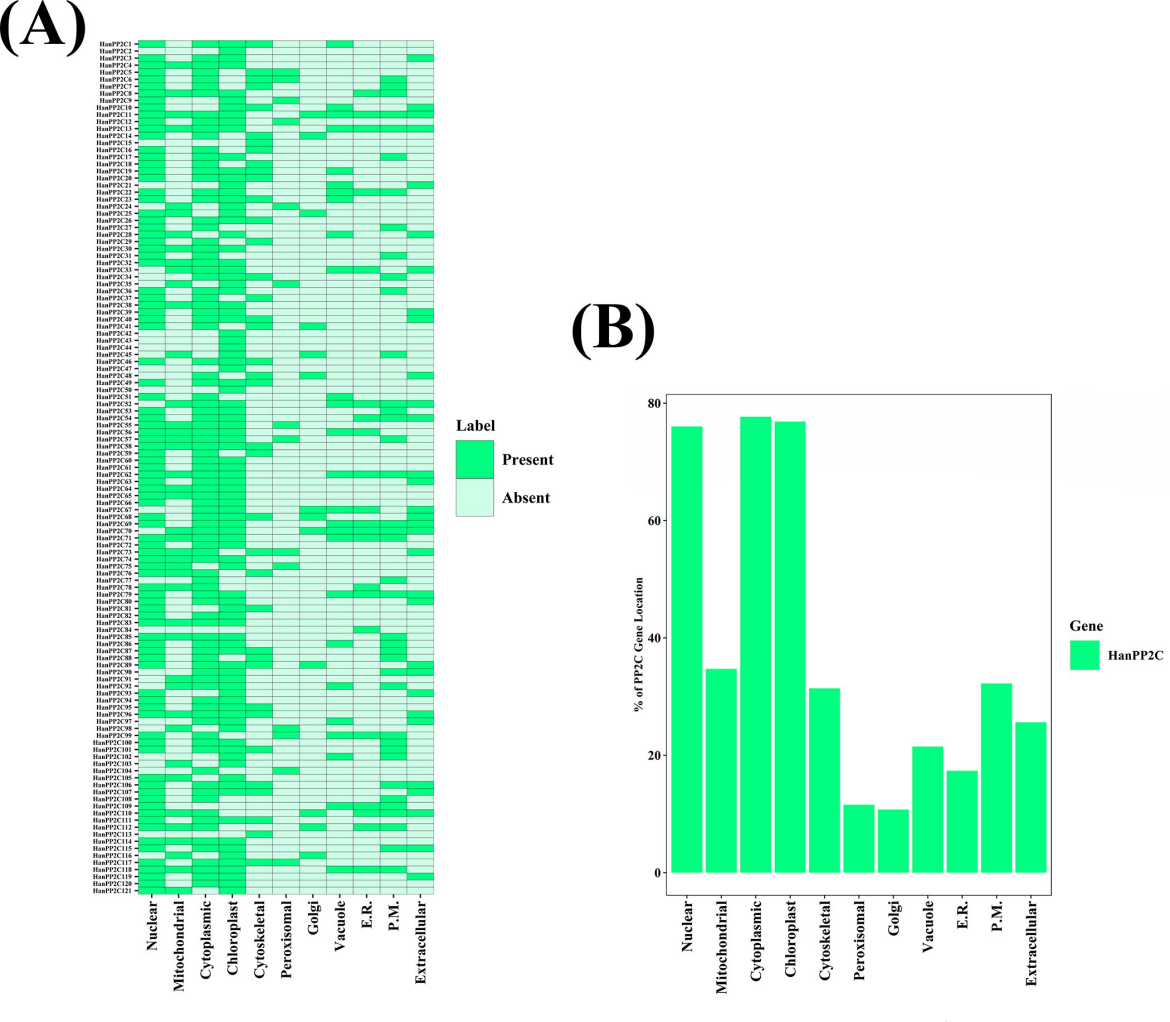
**(A) A heatmap represents the sub-cellular localization analysis of sunflower PP2C proteins.** The names of each HanPP2C protein are shown on the left side of the heatmap, while the terms of the corresponding cellular organelles are shown at the bottom of the heatmap. The intensity of color on the right side of the heatmap indicates the presence of protein signals corresponding to the genes. The cellular organelles include nuclear, mitochondrial, cytoplasmic, chloroplast, cytoskeletal, peroxisomal, Golgi, vacuole, endoplasmic reticulum (E.R.), plasma membrane (P.M.), and extracellular locations. **(B) The percentage distribution of sunflower PP2C protein signals across various cellular organelles is represented by a bar diagram.** The percentages of protein signals appearing in different cellular organelles are shown on the left side of the diagram. These organelles include nuclear, mitochondrial, cytoplasmic, chloroplast, cytoskeletal, peroxisomal, Golgi, vacuole, endoplasmic reticulum (E.R.), plasma membrane (P.M.), and extracellular locations.

Proteins are distributed throughout cells, which play a vital role in various physiological processes. The prediction of protein localization sites for the HanPP2C gene family can be demonstrated through subcellular localization prediction, thereby facilitating the analysis of gene functions. The subcellular analysis revealed that the HanPP2C protein signals were localized in various cellular organelles, including the nucleus, chloroplast, cytoplasm, mitochondria, cytoskeleton, peroxisome, Golgi apparatus, vacuole, endoplasmic reticulum, plasma membrane, and extracellular space (Fig 6A). Among these, the highest prediction sites for members were in the cytoplasm at 77.69% (94), followed by the chloroplast at 76.86% (93) and the nucleus at 76.03% (92). Conversely, the lowest protein sites were observed in the Golgi apparatus at 10.74% (13) and the peroxisome at 11.57% (14) among all predicted organelles (Fig 6B). Based on these findings, HanPP2C proteins exhibited organelle-specific localization and could function in different microenvironments. The subcellular localization prediction indicated that most HanPP2C genes may be located within organelles such as chloroplast, nucleus, cytoplasm, and mitochondria, while some might be found in the extracellular space. These results revealed the organelle-specific nature of HanPP2C genes and their functional diversity in various cellular contexts.

### 3.10 Cis-acting element analysis in the promoter regions

**Fig 7:**
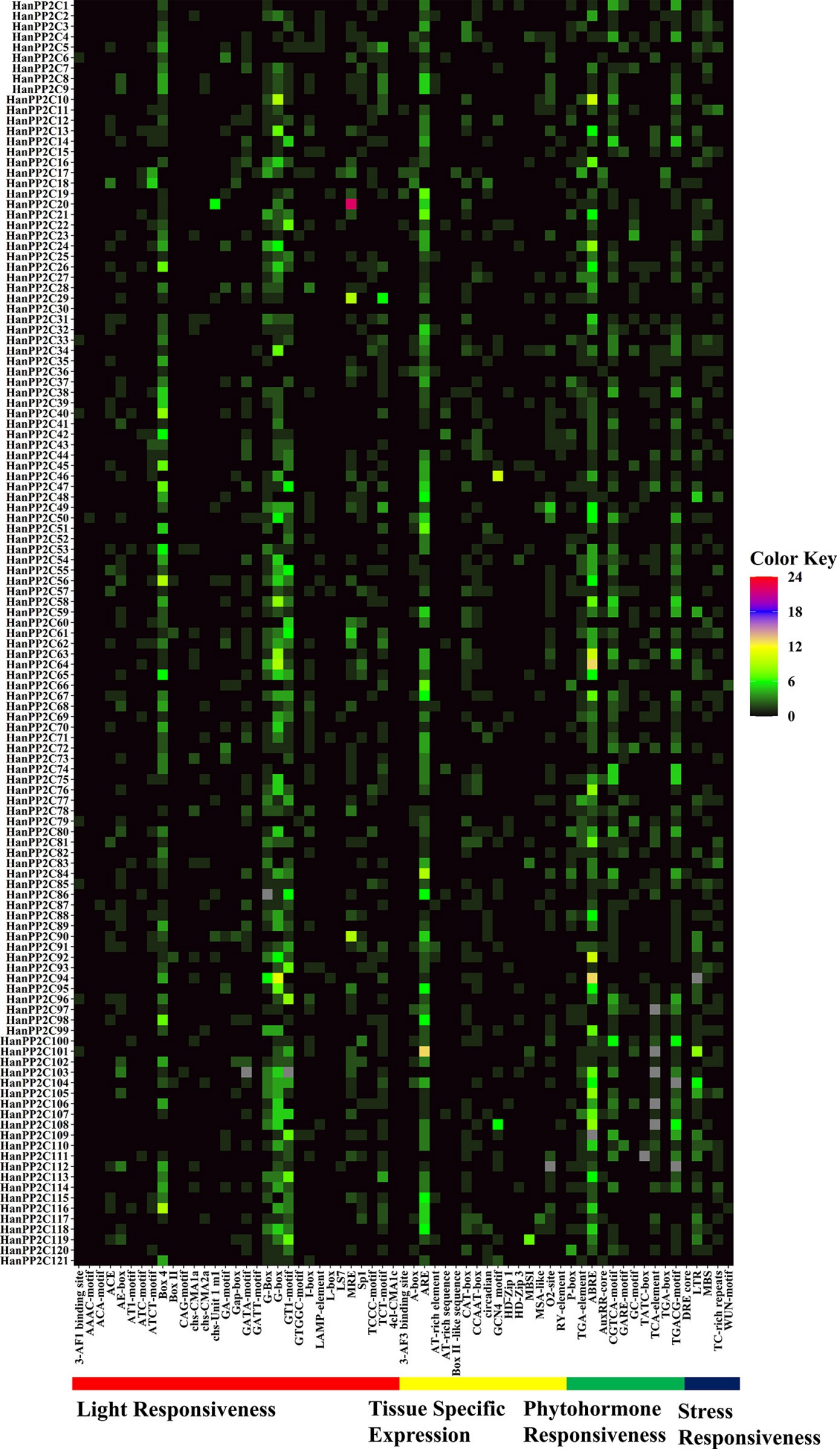
The distribution of putative cis-acting regulatory elements on the 2.0 kb promoter region of sunflower PP2C genes is represented by a heatmap. The names of each HanPP2C gene are shown on the left side of the heatmap. The number of putative cis-acting elements for each HanPP2C gene is displayed on the right side of the heatmap and is represented by four different colors (black = 0, green = 1–6, yellow = 7–12, purple = 13–18, and red = 19–24). Functions associated with cis-acting elements of the corresponding genes, such as light responsiveness, tissue-specific expression, phytohormone responsiveness, and stress responsiveness, are shown at the bottom of the heatmap and denoted by bold lines in red, yellow, green, and blue, respectively.

Cis-acting elements in the promoter region play a crucial role in achieving cell-specific, temporal, and spatial control over protein expression. This validates that gene promoters demonstrating similar expression patterns also contain comparable regulatory elements. The interaction between transcription factors and the cis-acting elements in the promoter region regulates gene transcription levels. In our analysis, we screened 63 cis-regulatory elements, including those responsive to light, tissue-specific, phytohormone, and stress, using the 2000 bp upstream region of the 5′‐UTR sequence of HanPP2C genes, utilizing the Plant CARE database (Fig 7 and S8 and S9 Data). Of these 63 cis-acting elements, 31 were associated with light responsiveness, 16 with tissue-specific expression, 11 with phytohormone responsiveness, and 5 with stress responsiveness.

The light responsive elements such as MRE, Box-4, G-box were found in abundance in the HanPP2C promoter region. Among them, MRE motif was highly expressed in HanPP2C20 followed by HanPP2C29 and HanPP2C90 containing 22, 09, and 09 MRE elements, respectively while Box-4 and G-box showed higher expression pattern in most HanPP2C genes. The tissue-specific responsive, ARE element was found abundantly in HanPP2C84, and HanPP2C101, containing 09 and 13 ARE elements. Furthermore, ABRE was the most abundant phytohormone-responsive cis-element in the HanPP2C promoter region. Among all HanPP2Cs, HanPP2C64 and HanPP2C94 contained the most significant number (13) of ABREs, followed by HanPP2C10 (10), HanPP2C63 (10), and HanPP2C92 (10)., respectively. Among stress-responsive cis-elements, LTR was highly expressed in HanPP2C48, HanPP2C101, and HanPP2C104 containing 05, 08, and 06 LTR elements, respectively. In summary, cis-regulatory elements responsible for light-specific, tissue-specific, phytohormone, and stress response were found abundantly in the promoter region of HanPP2C genes, with these elements ranging from 0 to 22. Therefore, analyzing the cis-elements of PP2C genes in sunflowers can help to identify the functions of HanPP2C genes.

### 3.11 Putative microRNA target site analysis

The Plant miRNA Encyclopedia (http://pmiren.com/) database was utilized to retrieve microRNA sequences targeting HanPP2C genes [48]. Seventy-one miRNAs were identified, targeting 86 of the 121 HanPP2C genes (S10 Data). None of these miRNAs were found to target the remaining 35 HanPP2C genes. The retrieved miRNAs ranged from 20 to 22 nucleotides, and the number of miRNAs targeting each HanPP2C gene varied from 1–13. Based on our observations, Han-miR172 (27), Han-miR156 (19), Han-miR167 (19), and Han-miR170 (14) were identified as highly abundant miRNAs, each comprising 10 (a-j), 13 (a-m), 5 (a-e) and 7 (a- c, e- h) members, respectively (Table 3). Han-miR172 was found to target six HanPP2C genes: HanPP2C22, HanPP2C38, HanPP2C64, HanPP2C67, HanPP2C71, and HanPP2C118, with HanPP2C22 being particularly targeted by nine types of Han-miR172. In contrast, Han-miR156 targeted four HanPP2C genes, namely HanPP2C14, HanPP2C70, HanPP2C100, and HanPP2C114, with HanPP2C14 being the most highly targeted gene, being targeted by seven types of Han-miR156. Moreover, Han-miR167 targeted four HanPP2C genes such as HanPP2C15, HanPP2C61, HanPP2C72, and HanPP2C84, while seven HanPP2C genes including HanPP2C10, HanPP2C19, HanPP2C32, HanPP2C67, HanPP2C78, HanPP2C95, and HanPP2C111 were found to be targeted by Han-miR170. This finding can be useful for understanding gene regulation and the responsiveness of sunflowers HanPP2C gene family to various stresses.

**Table 3:** Information about abundant miRNA ID, functions, and their targeted HanPP2C genes.

miRNA ID	Functions	Targeted genes	References
Han-miR172	Role in flowering time controlling, plant transitional stages, and plant shifting from vegetative to reproductive stages, controlling stem cell fate of flower meristem and photoperiods	HanPP2C22, HanPP2C38, HanPP2C64, HanPP2C67, HanPP2C71, HanPP2C118	[57–61]
Han-miR156	Act as a multi-stress response integrator	HanPP2C14, HanPP2C70, HanPP2C100, HanPP2C114,	[62, 63]
Han-miR167	Functions in regulating plant reproduction, gene expression, auxin response, as well as plant growth and development	HanPP2C15, HanPP2C61, HanPP2C72, HanPP2C84	[64]
Han-miR170	Role in response to drought stress, signaling pathway that promotes cell elongation in root developmental stages	HanPP2C10, HanPP2C19, HanPP2C32, HanPP2C67, HanPP2C78, HanPP2C95, HanPP2C111	[65, 66]

### 3.12 Transcriptomic expression pattern analysis in different tissues and stresses

**Fig 8:**
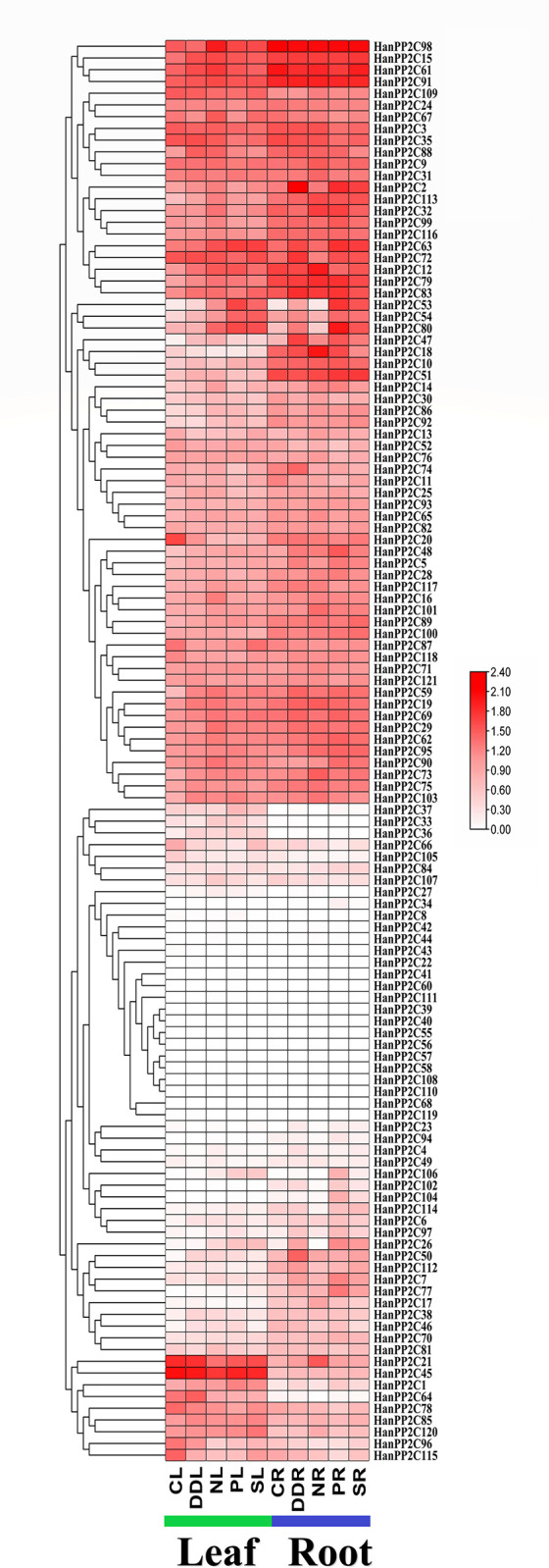
Expression profiles of HanPP2C genes in leaf and root tissues under different abiotic stress treatments. The clustering of HanPP2C genes in leaf (CL = Control, DDL = Dry Down, NL = Low nutrient, PL = PEG, and SL = Salt) and root tissue (CR = Control, DDR = Dry Down, NR = Low nutrient, PR = PEG, and SR = Salt) is based on their expression profiles under different abiotic stress treatments [50]. The FPKM values were transformed into the Log2 format and compared with the control. The expression values were clustered and visualized using TBtools version-v1.116 [41]. The color gradient from low to high expression (white to red color) is shown on the right side of the heatmap.

Differential expression profiles of 121 differentially expressed genes (DEGs) of HanPP2C genes at various developmental phases were investigated across four different treatments (control + four stresses) and two different types of tissues (leaf and root) compared to the control, using previously generated RNA-seq data (Fig 8) [50]. The expression patterns of HanPP2C genes were illustrated as a heatmap showing various expression levels on tissues and stresses. Among the identified HanPP2C genes, 47 HanPP2C genes exhibited differential expression in leaf tissue, while 49 HanPP2C genes displayed differential expression in root tissue under various stress conditions compared to the control. Additionally, 23 HanPP2C genes in leaf tissue and 22 HanPP2C genes in root tissue were unexpressed in any treatment, suggesting potential functions in other developmental stages.

The most DEGs (32) were observed in leaf tissue in the nutrient stress treatment. HanPP2C12, HanPP2C16, HanPP2C80, HanPP2C88, HanPP2C98 exhibited high expression levels, while HanPP2C19, HanPP2C29, HanPP2C59, HanPP2C63, HanPP2C69, HanPP2C73, HanPP2C90, HanPP2C103 were moderately expressed. In contrast, HanPP2C14, HanPP2C25, HanPP2C26, HanPP2C32, HanPP2C36, HanPP2C38, HanPP2C46, HanPP2C47, HanPP2C50, HanPP2C53, HanPP2C54, HanPP2C61, HanPP2C62, HanPP2C67, HanPP2C73, HanPP2C83, HanPP2C106, HanPP2C107, and HanPP2C113 genes showed low expression levels based on the heatmap. HanPP2C12, HanPP2C53, HanPP2C63, and HanPP2C80 were highly expressed in the PEG stress treatment, while HanPP2C53, HanPP2C63, and HanPP2C80 exhibited high expression levels in the salt treatment. Additionally, HanPP2C64, HanPP2C103, and HanPP2C88 were moderately expressed in the dry-down treatment.

In root tissue, the highest number of DEGs (25) was observed in the PEG stress treatment. HanPP2C7, HanPP2C48, HanPP2C53, HanPP2C63, HanPP2C77, HanPP2C80, and HanPP2C106 exhibited higher expression levels in this treatment. Under dry-down, nutrition stress, and salt treatment, 17, 17, and 13 DEGs exhibited varying expression levels, respectively. Five genes (HanPP2C2, HanPP2C47, HanPP2C50, HanPP2C72, and HanPP2C74) showed the highest expression levels in the dry-down treatment. In the nutrition stress treatment, HanPP2C18, HanPP2C12, HanPP2C21, and HanPP2C73 were highly expressed, while under salt treatment, HanPP2C2, HanPP2C53, HanPP2C63, and HanPP2C80 exhibited the highest expression levels. Among the 121 HanPP2C genes with differential expression levels, nine genes (HanPP2C12, HanPP2C36, HanPP2C38, HanPP2C47, HanPP2C48, HanPP2C53, HanPP2C54, HanPP2C59, and HanPP2C73) in leaf tissue and five HanPP2C genes (HanPP2C13, HanPP2C47, HanPP2C48, HanPP2C54, and HanPP2C95) in root tissue were expressed under all four stress treatments compared to the control. The differential expression levels of HanPP2C genes suggest distinct roles and functions in response to various treatments in leaf and root tissues.

### 3.13 Homology-based modeling of HanPP2C proteins

**Fig 9:**
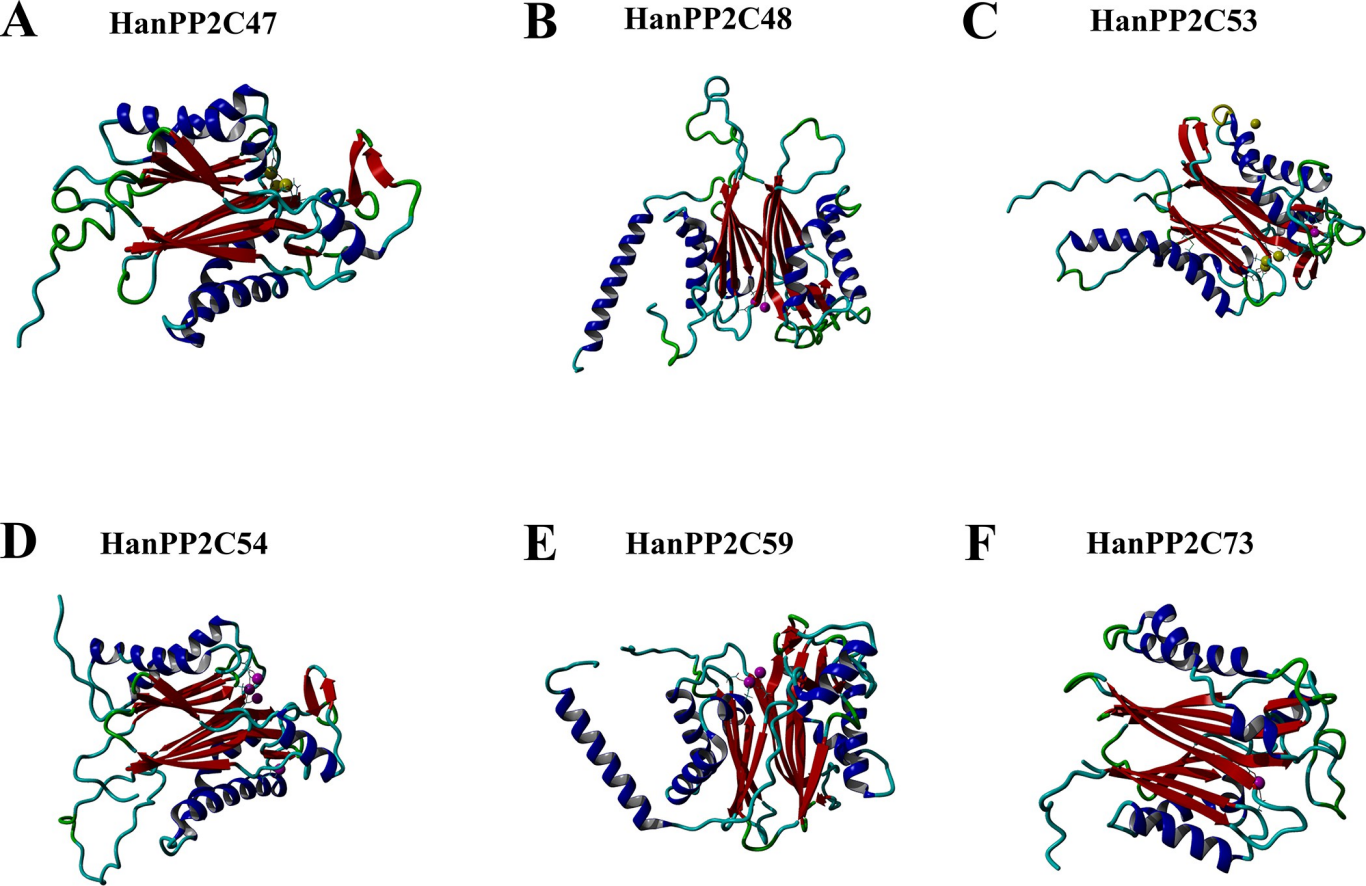
Three-dimensional (3D) homology-based models of sunflower PP2C proteins. Three-dimensional homology-based models of selected sunflower PP2C proteins with their predicted co-factor visualized using the YASARA homology software (version 22.9.24.W.64) [53]. The panels represent A. HanPP2C47 with predicted co-factor (pea green spheres), B. HanPP2C48 with predicted co-factor (purple spheres), C. HanPP2C53 with predicted co-factor (pea green spheres), D. HanPP2C54 with predicted co-factor (purple spheres), E. HanPP2C59 with predicted co-factor (purple spheres), F. HanPP2C73 with predicted co-factor (purple spheres).

The prediction of the 3D homology modeling of a protein structure is widely used due to its reliability, sensitive, time- saving, cost-effectiveness, and rapidity when compared with NMR or X-ray diffraction analyses. This alternative emerges from the advancement of *in silico* analysis tools [67–69]. Further, based on the transcriptomic expression analysis under different stress conditions, a total of six HanPP2C proteins (HanPP2C47, HanPP2C48, HanPP2C53, HanPP2C54, HanPP2C59, and HanPP2C73) were selected to construct the 3D homology models (Fig 9). Additionally, the selection of predicted homology modeling templates was based on higher total Z-scores. In this analysis, 3D structures of six candidate HanPP2C proteins were predicted, providing acceptable models.

## 4.0 Discussion

Following the completion of the sunflower (*H*. *annuus*) whole genome sequencing, several important gene families have been identified at genome level, including the PM H^+^-ATPase gene family [70], WRKY [71], MAPK [72], OSCA [73], WSD [74], VQ [75], Threlix transcription factor [76], NBS-LRR [77], NAC-TF [78]. In our analysis, we identified a total of 121 genes in sunflower, which was higher than Arabidopsis (80) [10], cucumber (56) [56], rice (78) [10], and maize (97) [79]. This observation suggests that the HanPP2C gene family members are the largest compared to other plant species. Notably, the expansion of PP2C genes varies among species and may be relevant to their adaption to stressful environmental conditions.

According to phylogenetic analysis, the members of the PP2C protein family were classified in 13 subgroups in several plants species such as *A*. *thaliana* [10], rice [10], cucumber [56], and wheat [14]. Moreover, in woodland strawberries, and pineapple strawberries, PP2C proteins were clustered into 11 subgroups [80]. However, HanPP2C were clustered into 15 subgroups showing quite dissimilarity to the above species. Additionally, FaPP2C and FvPP2C in woodland, and pineapple strawberries were identified in 10 and 9 subgroups out of 11 subgroups, respectively [80]. Similar findings were observed in this study where HanPP2C proteins were present in 14 out of 15 subgroups except for subgroup K. The presence of HanPP2C and AtPP2C genes within each subfamily, closely linked to genes of the same species, implies the existence of an ancestral set of genes defining each subfamily before the monocot-eudicot separation. Furthermore, the phylogenetic structure of the PP2C family aligns well with the concept of birth and death evolution within the flowering plant lineage [81, 82]. Branches containing more than one HanPP2C or AtPP2C gene likely resulted from gene duplication, whereas branches containing only HanPP2C or AtPP2C genes probably experienced gene loss. Similar birth and death evolution patterns are observed in other gene families, such as MADS-box, involved in plant flower development [83]. The phylogenetic tree results indicate that the closer the grouping, the higher the likelihood of having similar functions. Our findings suggest a consistent paralogous sequence for HanPP2C gene divergences through gene duplication.

We analyzed the gene structure of the HanPP2C family, a crucial indicator of the ancestral relationships among all members of the targeted gene family [84]. The gene structure can be valuable for exploring evolutionary links among organisms or genes [85]. According to our findings, HanPP2C genes within the same group exhibited the same exon-intron structure having 1–20 exons, although some variation in the exon-intron distribution pattern was observed, which can be attributed to various factors. Similar number of exons were previously identified in PP2C gene family of tomato [31].Gene structure patterns of HanPP2C showed the similarities with the PP2C genes of Arabidopsis [10], rice [10], cucumber [56], and woodland and pineapple strawberries [80], indicated the conservation of these gene structures throughout evolution [86]. However, our results showed quite differences in exon-intron number from above species. The intron numbers of PP2C genes in Arabidopsis, rice, woodland strawberries and pineapple strawberries ranged from 0–12, 0–18,0–14, 0–34, respectively [10, 80]. Our findings align with the previous investigations revealing the presence of intron-less genes in PP2C family. The individual genes in the sunflower PP2C family showed structural differences, and the variable exon-intron structure contributes to the diversity of gene functions.

We conducted the domain analysis to detect the types and numbers of conserved domains within the HanPP2C gene family. Among all the domains, the PKc domain was also appeared in Barley PP2C genes along with CAP_ED, PKc_like, MSCRAMM_ClfB, NB-ARC, PLN03200, Arm, LRR and Rx_N domains [87]. The PKc domain contains phosphorylation binding sites, is highly conserved and acts as a converter of external signals into secondary signals within plant cells [88]. Under salt stress conditions, the PKc domain of receptor-like-kinase (RLK) signaling pathways may initiate signal transduction by phosphorylating target proteins or kinases [89]. Further, FHA (forkhead associated domain) was found in *Brachypodium distachyon* PP2C genes along with S-TKc (ser/thr kinase catalytic domain), and CNB (cyclic nucleotide-binding domain) [90]. The FHA domain is a phosphothreonine recognition module in various signaling proteins, including Arabidopsis kinase-associated protein phosphatase (KAPP). KAPP’s kinase-interacting FHA (KI-FHA) domain functions as a negative regulator in several RLK signaling pathways involved in plant growth, development, and responses to environmental stresses [91].

Furthermore, the diverse distribution of motifs in protein sequences can serve as a potential indicator of the divergence of gene functions within different subgroups. Proteins in the same subgroup demonstrate similar motif distributions, suggesting a close evolutionary relationship. However, few genes lacked specific motifs, and these differences in motif composition may cause functional diversity. Previous studies on motif distribution in Arabidopsis [10], rice [10], and cucumber [56] PP2C genes demonstrated that motifs were particular to only one or more subgroups which support our findings. Notably, the number of identified conserved motifs in HanPP2C (20 motifs) is higher than cucumber (10 motifs) [56], tomato (10 motifs) [31], peanut (10 motifs) [92], Arabidopsis (11 motifs) [10], and *Medicago truncatula* (15 motifs) [9].

Evaluating selective pressure offers valuable guidance for identifying amino acid sequences within a protein. It is also necessary for analyzing functional residues and structural protein shifts [93]. This study revealed that all 53 pairs of duplicated HanPP2c genes predominantly evolved through purifying selection. Previous investigation on tomato [31] and peanut [92] PP2C genes showed that all duplicated SIPP2C and AhPP2C genes have evolved from purifying selection. However, both purifying and positive selection were identified in the duplicated PP2C genes of woodland strawberries and pineapple strawberries [80]. Furthermore, Ks values were analyzed to determine the divergence period, and the divergence period of these paralogous genes ranged from 0.02 to 0.63 (Ks values) with an average duplication time of 21.72 MYA. These results suggest that they diverged more recently than Arabidopsis (16.10 MYA) [11] and at a faster rate than tomato (31.59 MYA) [31].

The collinearity analysis revealed the presence of 53 homologous HanPP2C gene pairs and suggested that the abundance of PP2C genes in sunflower can be attributed to whole-genome duplication. Collinearity and chromosomal distribution indicated a significant role for segmental duplication in the abundance of the HanPP2C gene family, which is similar to previous findings in rice [10], Arabidopsis [10], and cucumber [56]. It is noteworthy that the number of collinear gene pairs of HanPP2C family is higher than tomato (17 pairs) [31], Arabidopsis (9 pairs) and rice (3 pairs) PP2C family [31]. Moreover, we identified syntenic correlations in 10 PP2C gene pairs between sunflower and Arabidopsis The results of the synteny analysis can be used to highlight the functional and evolutionary relationship between sunflower and Arabidopsis species. However, 12, 6, 5 PP2C gene pairs were previously represented as syntenic pairs between tomato-Arabidopsis, tomato-rice, Arabidopsis-rice, respectively [31]. Our study revealed a higher degree of homology between the HanPP2C and AtPP2C gene families, consistent with previous findings between Arabidopsis and cucumber [56], which showed 59 syntenic gene pairs between 48 AtPP2Cs and 41 CsPP2Cs, further supporting the conception of more significant homology between AtPP2C and CsPP2C genes.

According to chromosomal localization, HanPP2C genes were unevenly scattered across 17 chromosomes while CsPP2C, MtPP2C, OsPP2C and AhPP2C genes of cucumber [56], *Medicago truncatula* [9], rice [10] and peanut [92] were distributed across 7, 8, 12 and 20 chromosomes, respectively [9, 56]. Chromosomal localization of genes indicates gene duplication, a significant driving force in biological evolution, and it can lead to HanPP2C variation [94]. The HanPP2C genes expand through both segmental and tandem duplication, although tandem duplications are less prevalent. and segmental duplication predominated over tandem duplication. This results align with the previous investigation on the PP2C genes of rice representing a total of 12 segmental and 4 tandem duplicated gene clusters [10]. However, no tandem duplications have been reported in the PP2C gene families of *Arachis hypogaea* [92], *Brachypodium distachyon* [90], *Medicago trauncatula* [9] *Cucumis sativus* L [56]. Gene duplication plays a primary role in gene expansion, and the increasing number of HanPP2C genes in higher plants may be attributed to domain duplication throughout eukaryotic plant evolution [95]. In the gene replication process, segmental duplication is more favorable for maintaining gene function than tandem repeats [96].

The prediction of subcellular localization helps investigate the roles of gene families more precisely and conveniently. HanPP2C genes were highly expressed in the cytoplasm, chloroplast and nucleus. The PP2C genes of barley [87] and tomato [31], primarily localized in the chloroplast, cytosol and nucleus. In cucumber [56], *Brachypodium distachyon* [90], *Medicago trauncatula* [9], woodland strawberries and pineapple strawberries [80] PP2C genes were also highly present in the nucleus, chloroplast, and cytoplasm, consistent with our findings. The subcellular localization prediction suggests that proteins located in the cytoplasm are mainly involved in the cytoskeleton formation, acting as actin regulators. The differential localization of proteins within distinct subcellular compartments indicates significant function differences [97, 98]. Understanding protein subcellular localization provides crucial information for determining protein biological activity. Our analysis shows that HanPP2C genes are likely involved in respiration, photosynthesis, cellular growth, and development processes, as they were predominantly localized in the cytoplasm, chloroplast, and nucleus.

Cis-regulatory elements within gene promoter upstream regions play vital roles in plant stress responses. For example, ABA-responsive elements (ABREs) respond to ABA, dryness, or salt signals [99], while LTR is an essential element for low-temperature regulation and stress responsiveness [100]. The analysis of cis-regulatory elements in the 121 HanPP2C genes revealed the presence of one or more ARE, ABRE, G-Box, MRE, GTI, LTR, MRE, DRE, and other cis-acting elements, indicating a significant link between HanPP2C genes and plant stress responses. Among these, light responsive elements comprised a significant portion of HanPP2C genes, demonstrating the photo-regulated activity of PP2C proteins in sunflower. Light-responsive elements may play a crucial role in the photosynthetic mechanism of sunflower, potentially enhancing productivity, and grain quality [101]. Furthermore, various plant biological processes are associated with predicted tissue-specific motifs such as ARE, CAT-box, AT-rich elements, MBS 1-motif, MSA-like elements, HD-Zip 1, and HD-Zip 3. Plant hormones, also known as growth regulators, have regulatory functions in the growth, development, metabolism, and seed germination activities [102, 103]. In this study, TGA- elements, ABRE, TCA-elements, TATC-box, and GC-motif were predicted in phytohormone responsiveness, responsible for various biological functions involved in the growth and development of sunflower. Moreover, LTR, DRE, MBS, TC-rich repeats, and WUN motifs control the expression of genes associated with stress responsiveness, enabling plants to adapt to harsh conditions [104, 105]. The type 2 protein phosphatase, named HIGHLY ABA-INDUCED PP2C1 (HAI1) interacted with FL7 (FORKED-LIKE7), where HAI1 was suppressed. FL7 enhanced the plant immunity response through the phosphorylation of MPK3 (MITOGEN-ACTIVATED PROTEIN KINASE 3) and MPK6 [106]. Our results showed similarity with previous findings that ABREs (involved in ABA responsiveness) are abundant in the promoter regions of PP2C genes in Arabidopsis and rice [10]. In woodland strawberries, most PP2C genes contain ABA-responsive elements [80]. Moreover, ARE, LTR, MBS, TC-rich repeat ABRE, CGTCA-motif, TATC-motif, TCA element, and TGA-element were also identified as highly expressed cis-regulatory elements in *Medicago truncatula* consistent with our findings [9].

MicroRNAs play a crucial role as plant regulators, regulating various biological processes, including plant growth and development [107]. Numerous miRNAs have recently been found in various species such as soybean (*Glycine max*) [108], *Brassica napus* [109, 110], *Arachis hypogaea* [111], maize (*Zea mays*) [112] involved in various developmental and biological processes as well as stress response mechanisms of species. The number of HanPP2C genes targeted miRNA (71 different miRNAs) is significantly higher than AhPP2C genes targeted miRNA of peanut (14 different miRNAs) consisting 5 common miRNAs (miR156, miR159, miR167, miR408, and miR1516) [92]. Among these, the highly abundant miR172 of HanPP2C family has various functions, such as controlling flowering time, transitioning between different plant growth stages, and shifting from vegetative to reproductive stages [57, 58]. In Arabidopsis, miR172 plays a vital role in controlling stem cells’ fate, flowering time, and responding to photoperiod changes [59–61]. The other miRNA, miR156, shows versatile functions in different plant developmental stages and is an essential integrator to respond to multiple stresses [62, 63]. Previous research has looked into the functions of miR156 in response to drought, salt, and cold stresses through microRNA sequencing [113]. In *Triticum aestivum*, miR156 has increased plant susceptibility to heat stress [114]. Similarly, miR167, another abundant microRNA, primarily regulates plant reproduction [64]. In *Oryza sativa*, miR167 plays a crucial role in gene expression, auxin response, and overall plant growth and development. The role of miR170 has been exhibited to be induced in response to drought stress in plants [65]. The miR170/SCL (transcription factor) node is involved in the gibberellin signaling pathway, promoting cell elongation during root developmental stages [66]. This suggests the potential involvement of HanPP2C genes in promoting vegetative growth while limiting reproductive and floral development and mediating different abiotic stress responses.

According to previously generated RNA-seq data, we observed significant differences in gene expression patterns in both root and leaf tissues in response to different stress conditions [50]. Furthermore, several HanPP2C genes demonstrated a consistent expression pattern across all four stress treatments, comparable with previous findings in cucumber [56]. Notably, nutrient stress induced the highest number of differentially expressed genes in leaf tissue, suggesting a significant impact on leaf mass fraction (LMF). Conversely, no phenotypic divergence was observed between the control samples and PEG stressed in root tissues, although, PEG stress resulted in the highest number of expressed genes in root tissues. However, many genes were either silenced or showed lower expression levels. In this study, drought stress in leaf tissues and salinity stress in root tissues suppressed the expression of most HanPP2C genes, indicating their potential role as negative regulators. In wheat, TaPP2C59 was also suppressed by salinity stress and ABA signaling, suggesting it may act as a negative regulator in abiotic stress and ABA signaling [115]. Additionally, in maize, the ZmPP2C genes act as negative regulators of drought and salinity stress [116]. However, HanPP2C genes that were highly expressed under all four treatments may be speculated as positive regulators. Previous studies in Arabidopsis identified several AtPP2C genes as positive regulators of salt tolerance and drought stress in peaches [117, 118]. Under salt stress, Na^+^ is transported out of the cell through the activity of a protein phosphatase. PP2C activates SOS1 (the salt overly sensitive pathway) in corporated with SOS2 to increase salt tolerance in plants [119]. PtPP2C genes in *Populus euphratica* exhibited a higher expression pattern under cold, drought, and high salt stress [120]. VvPP2C02 in grapes strongly responded to high salt, drought, and ABA signaling [121]. The expression patterns of HanPP2C under all four treatment align with the findings of cucumber [56] and *Medicago trauncatula* [9]. These findings serve as a foundation for further research on the functions and expression patterns of HanPP2C genes under various stress conditions.

Homology modeling investigations are employed to solve multiple challenges related to protein crystallization, thus providing a pathway to gaining higher structural insights into proteins through *in-silico* approaches [122]. The results presented here are recommended as the first homology structural predictions for the HanPP2C protein family, which includes HanPP2C47, HanPP2C48, HanPP2C53, HanPP2C54, HanPP2C59, and HanPP2C73. The homology model of HanPP2C47 showed structural similarity to P2C56_ARATH, a key negative modulator of the abscisic acid (ABA) signaling pathway in *A*. *thaliana* [123, 124]. The structural model of HanPP2C48 is closely similar to the homology model of PPM1A_HUMAN Protein phosphatase 1A (PP1A) of humans (*Homo sapiens*), which exhibited broad specificity [125]. HanPP2C53 homology model template closely resembled P2C37_ARATH of *A*. *thaliana*, another negative regulator of ABA responses across biological activities [126]. The 3D homology model of HanPP2C54 demonstrated a close similarity to PP2C6 (2C68_ORYSJ) of rice (*Oryza sativa*), which is associated with the regulation of different abiotic stress responses [127]. The homology model of HanPP2C59 remarkably resembles human PPM1B proteins, a member of the PP2C family of Ser/Thr protein phosphatases. Previous studies have shown that PP2C family members act as negative regulators of cell stress response pathways [128]. However, the predicted homology model of HanPP2C73 also exhibited similarity to the model of Arabidopsis P2C56_ARATH proteins, similar to the HanPP2C47 model. These structural models of candidate HanPP2C proteins could provide valuable insights into understanding the significant biological functions associated with phosphatase activity at the molecular level. However, further detailed studies are suggested to identify the factors regulating phosphatase activity.

## 5.0 Conclusion

In the present study, we identified 121 HanPP2C genes throughout the sunflower genome, employing a comprehensive bioinformatics analysis distributed across the 17 chromosomes. We observed segmental duplication in 48 gene pairs among the total 53 pairs. The gene structures, conserved domains, and motifs of these 121 HanPP2C genes exhibited remarkable similarity. Analysis of selection pressure and collinearity suggested that HanPP2C genes evolved through purifying selection, maintaining functional stability. Further, cis-acting elements analysis revealed the presence of regulatory elements associated with response to light, tissue specificity, phytohormone, and stress. The expression profiles of the HanPP2C gene family members display varying responses to multiple abiotic stresses including dry conditions, low nutrient availability, PEG-induced stress, and salt treatments, compared to the control conditions. The homology modeling of six candidate HanPP2C proteins provide valuable information to understand their biological functions at molecular level. The findings provide useful insight for future research on stress mechanisms, gene selection, gene function elucidation, and the development of stress-tolerant sunflower cultivars in future breeding programs.

## Supporting information

S1 Data Full-length protein sequences of PP2C gene families of *A*. *thaliana* and *H*. *annuus* plant species for constructing a phylogenetic tree. (TXT)

S2 Data Full-length coding sequences of HanPP2C gene families of *H*. *annuus* plant species. (TXT)

S3 Data Full-length genomic sequences of HanPP2C gene families of *H*. *annuus* plant species. (TXT)

S4 Data Full-length protein sequences of HanPP2C gene families of *H*. *annuus* plant species. (TXT)

S5 Data Sunflower PP2C gene family distribution among groups based on phylogenetic analysis with Arabidopsis PP2C members. (DOCX)

S6 Data *In silico* predicted the number of introns and exons in HanPP2C genes. (DOCX)

S7 Data Time of gene duplication estimated for different paralogous pairs of HanPP2C genes based on Ka and Ks values. (XLSX)

S8 Data The upstream promoter region (2.0 kb genomic sequences) of HanPP2C gene families of *H*. *annuus* for analysis of cis-acting regulatory elements. (TXT)

S9 Data The predicted cis-acting regulatory elements of the upstream promoter region (2.0 kb genomic sequences) of HanPP2C gene families of *H*. *annuus*. (XLSX)

S10 Data miRNA targeted prediction of HanPP2C. The miRNA data was downloaded from the plant micro-RNA encyclopedia (http://pmiren.com/).
(DOCX)

S1 Fig The sequence logos of 20 motifs present in *H*. *annuus* HanPP2C proteins. (TIF)

S2 Fig The chromosomal locations and duplications of sunflower HanPP2C genes. The number of distinct chromosomes is at the top of each chromosome bar. The chromosome-scale is in millions of bases (Mb), indicating the length of each chromosome on the left, using the information retrieved from Phytozome v13. Light orange lines indicate tandem duplications, while light blue lines indicate segmental duplications.
(TIF)
