# Supplementary material for: Genome-wide identification and characterization of protein phosphatase 2C (PP2C) gene family in sunflower (Helianthus annuus L.) and their expression profiles in response to multiple abiotic stresses
Source: PLoS One. 2024 Mar 20;19(3):e0298543. doi: 10.1371/journal.pone.0298543 (PMC10954154; doi:10.1371/journal.pone.0298543)
Supplement: S5 Data — (DOCX) [file pone.0298543.s005.docx]

**S5 Data. Sunflower *PP2C* gene family distribution among groups based on phylogenetic analysis with Arabidopsis PP2C members.**

| Group | Number of PP2C genes | Gene ID |
| --- | --- | --- |
| A1 | 8 | HanPP2C27, HanPP2C54, HanPP2C62, HanPP2C68, HanPP2C69, HanPP2C79, HanPP2C94, HanPP2C104 |
| A2 | 9 | HanPP2C26, HanPP2C34, HanPP2C39, HanPP2C40, HanPP2C41, HanPP2C53, HanPP2C63, HanPP2C80, HanPP2C119 |
| B1 | 7 | HanPP2C2, HanPP2C42, HanPP2C43, HanPP2C44, HanPP2C47, HanPP2C50, HanPP2C102 |
| B2 | 1 | HanPP2C46 |
| C | 7 | HanPP2C20, HanPP2C25, HanPP2C51, HanPP2C81, HanPP2C96, HanPP2C100, HanPP2C110 |
| D | 20 | HanPP2C1, HanPP2C8, HanPP2C13, HanPP2C17, HanPP2C28, HanPP2C60, HanPP2C65, HanPP2C71, HanPP2C74, HanPP2C75, HanPP2C86, HanPP2C87, HanPP2C90, HanPP2C92, HanPP2C99, HanPP2C101, HanPP2C103, HanPP2C114, HanPP2C116, HanPP2C118 |
| E | 14 | HanPP2C9, HanPP2C18, HanPP2C21, HanPP2C24, HanPP2C30, HanPP2C35, HanPP2C36, HanPP2C82, HanPP2C85, HanPP2C98, HanPP2C105, HanPP2C111, HanPP2C117, HanPP2C121 |
| F1 | 8 | HanPP2C16, HanPP2C32, HanPP2C73, HanPP2C77, HanPP2C83, HanPP2C88, HanPP2C108, HanPP2C113 |
| F2 | 5 | HanPP2C15, HanPP2C61, HanPP2C64, HanPP2C72, HanPP2C91 |
| G | 15 | HanPP2C4, HanPP2C5, HanPP2C6, HanPP2C7, HanPP2C14, HanPP2C23, HanPP2C29, HanPP2C48, HanPP2C52, HanPP2C59, HanPP2C66, HanPP2C89, HanPP2C97, HanPP2C107, HanPP2C112 |
| H | 8 | HanPP2C3, HanPP2C11, HanPP2C12, HanPP2C33, HanPP2C67, HanPP2C78, HanPP2C115, HanPP2C120 |
| I | 4 | HanPP2C10, HanPP2C19, HanPP2C31, HanPP2C95 |
| J | 6 | HanPP2C55, HanPP2C56, HanPP2C57, HanPP2C58, HanPP2C70, HanPP2C84 |
| L | 2 | HanPP2C38, HanPP2C109 |
| Outgroup | 7 | HanPP2C22, HanPP2C37, HanPP2C45, HanPP2C49, HanPP2C76, HanPP2C93, HanPP2C106 |
