# Supplementary material for: Genome-wide identification and characterization of protein phosphatase 2C (PP2C) gene family in sunflower (Helianthus annuus L.) and their expression profiles in response to multiple abiotic stresses
Source: PLoS One. 2024 Mar 20;19(3):e0298543. doi: 10.1371/journal.pone.0298543 (PMC10954154; doi:10.1371/journal.pone.0298543)
Supplement: S6 Data — (DOCX) [file pone.0298543.s006.docx]

**S6 Data. *In silico* predicted the number of introns and exons in HanPP2C genes.**

| **Group** | **PP2C gene** | **Source accession** | **Intron** | **Exon** |
| --- | --- | --- | --- | --- |
| A1 | HanPP2C27 | HanXRQChr05g0141351 | 3 | 4 |
|  | HanPP2C54 | HanXRQChr09g0238691 | 3 | 4 |
|  | HanPP2C62 | HanXRQChr09g0248001 | 3 | 4 |
|  | HanPP2C68 | HanXRQChr09g0276231 | 2 | 3 |
|  | HanPP2C69 | HanXRQChr09g0276631 | 3 | 4 |
|  | HanPP2C79 | HanXRQChr10g0312351 | 3 | 4 |
|  | HanPP2C94 | HanXRQChr13g0405491 | 3 | 4 |
|  | HanPP2C104 | HanXRQChr15g0486111 | 3 | 4 |
| A2 | HanPP2C26 | HanXRQChr05g0135271 | 3 | 4 |
|  | HanPP2C34 | HanXRQChr06g0179071 | 3 | 4 |
|  | HanPP2C39 | HanXRQChr07g0201631 | 1 | 2 |
|  | HanPP2C40 | HanXRQChr07g0201641 | 3 | 4 |
|  | HanPP2C41 | HanXRQChr07g0201651 | 3 | 4 |
|  | HanPP2C53 | HanXRQChr08g0237391 | 3 | 4 |
|  | HanPP2C63 | HanXRQChr09g0250051 | 3 | 4 |
|  | HanPP2C80 | HanXRQChr10g0312771 | 3 | 4 |
|  | HanPP2C119 | HanXRQChr17g0551771 | 4 | 5 |
| B1 | HanPP2C2 | HanXRQChr01g0011751 | 2 | 3 |
|  | HanPP2C42 | HanXRQChr07g0203201 | 3 | 4 |
|  | HanPP2C43 | HanXRQChr07g0203271 | 3 | 4 |
|  | HanPP2C44 | HanXRQChr07g0203281 | 3 | 4 |
|  | HanPP2C47 | HanXRQChr08g0214071 | 2 | 3 |
|  | HanPP2C50 | HanXRQChr08g0229111 | 2 | 3 |
|  | HanPP2C102 | HanXRQChr14g0459551 | 3 | 4 |
| B2 | HanPP2C46 | HanXRQChr08g0208311 | 4 | 5 |
| C | HanPP2C20 | HanXRQChr03g0080761 | 2 | 3 |
|  | HanPP2C25 | HanXRQChr05g0134881 | 3 | 4 |
|  | HanPP2C51 | HanXRQChr08g0234791 | 3 | 4 |
|  | HanPP2C81 | HanXRQChr11g0327411 | 3 | 4 |
|  | HanPP2C96 | HanXRQChr13g0411331 | 2 | 3 |
|  | HanPP2C100 | HanXRQChr13g0416691 | 3 | 4 |
|  | HanPP2C110 | HanXRQChr16g0507761 | 1 | 2 |
| D | HanPP2C1 | HanXRQChr01g0009741 | 4 | 5 |
|  | HanPP2C8 | HanXRQChr01g0027041 | 3 | 4 |
|  | HanPP2C13 | HanXRQChr02g0051821 | 3 | 4 |
|  | HanPP2C17 | HanXRQChr03g0068701 | 3 | 4 |
|  | HanPP2C28 | HanXRQChr05g0142971 | 3 | 4 |
|  | HanPP2C60 | HanXRQChr09g0246131 | 3 | 4 |
|  | HanPP2C65 | HanXRQChr09g0257251 | 3 | 4 |

**S6 Data** (Continued)

| **Group** | **PP2C gene** | **Source accession** | **Intron** | **Exon** |
| --- | --- | --- | --- | --- |
|  | HanPP2C71 | HanXRQChr10g0288231 | 5 | 6 |
|  | HanPP2C74 | HanXRQChr10g0297851 | 3 | 4 |
|  | HanPP2C75 | HanXRQChr10g0297981 | 3 | 4 |
|  | HanPP2C86 | HanXRQChr11g0349591 | 3 | 4 |
|  | HanPP2C87 | HanXRQChr11g0353861 | 3 | 4 |
|  | HanPP2C90 | HanXRQChr13g0390901 | 3 | 4 |
|  | HanPP2C92 | HanXRQChr13g0393511 | 3 | 4 |
|  | HanPP2C99 | HanXRQChr13g0414541 | 3 | 4 |
|  | HanPP2C101 | HanXRQChr14g0439281 | 3 | 4 |
|  | HanPP2C103 | HanXRQChr15g0484771 | 3 | 4 |
|  | HanPP2C114 | HanXRQChr16g0514931 | 3 | 4 |
|  | HanPP2C116 | HanXRQChr16g0531691 | 3 | 4 |
|  | HanPP2C118 | HanXRQChr17g0545241 | 3 | 4 |
| E | HanPP2C9 | HanXRQChr02g0032441 | 4 | 5 |
|  | HanPP2C18 | HanXRQChr03g0078771 | 4 | 5 |
|  | HanPP2C21 | HanXRQChr03g0083811 | 4 | 5 |
|  | HanPP2C24 | HanXRQChr04g0123011 | 4 | 5 |
|  | HanPP2C30 | HanXRQChr05g0148521 | 3 | 4 |
|  | HanPP2C35 | HanXRQChr06g0179631 | 4 | 5 |
|  | HanPP2C36 | HanXRQChr06g0182161 | 4 | 5 |
|  | HanPP2C82 | HanXRQChr11g0335211 | 4 | 5 |
|  | HanPP2C85 | HanXRQChr11g0340081 | 4 | 5 |
|  | HanPP2C98 | HanXRQChr13g0413441 | 4 | 5 |
|  | HanPP2C105 | HanXRQChr15g0492141 | 4 | 5 |
|  | HanPP2C111 | HanXRQChr16g0510191 | 4 | 5 |
|  | HanPP2C117 | HanXRQChr17g0542581 | 4 | 5 |
|  | HanPP2C121 | HanXRQChr01g0009741 | 4 | 5 |
| F1 | HanPP2C16 | HanXRQChr03g0068321 | 4 | 5 |
|  | HanPP2C32 | HanXRQChr05g0162881 | 4 | 5 |
|  | HanPP2C73 | HanXRQChr10g0295911 | 4 | 5 |
|  | HanPP2C77 | HanXRQChr10g0302681 | 4 | 5 |
|  | HanPP2C83 | HanXRQChr11g0338121 | 4 | 5 |
|  | HanPP2C88 | HanXRQChr12g0361131 | 4 | 5 |
|  | HanPP2C108 | HanXRQChr15g0494551 | 2 | 3 |
|  | HanPP2C113 | HanXRQChr16g0514801 | 4 | 5 |
| F2 | HanPP2C15 | HanXRQChr03g0065811 | 2 | 3 |
|  | HanPP2C61 | HanXRQChr09g0246191 | 0 | 1 |
|  | HanPP2C64 | HanXRQChr09g0253021 | 7 | 8 |
|  | HanPP2C72 | HanXRQChr10g0294661 | 0 | 1 |

**S6 Data** (Continued)

| **Group** | **PP2C gene** | **Source accession** | **Intron** | **Exon** |
| --- | --- | --- | --- | --- |
|  | HanPP2C91 | HanXRQChr13g0393121 | 7 | 8 |
| G | HanPP2C4 | HanXRQChr01g0025621 | 1 | 2 |
|  | HanPP2C5 | HanXRQChr01g0025691 | 1 | 2 |
|  | HanPP2C6 | HanXRQChr01g0025731 | 1 | 2 |
|  | HanPP2C7 | HanXRQChr01g0025741 | 1 | 2 |
|  | HanPP2C14 | HanXRQChr03g0063961 | 3 | 4 |
|  | HanPP2C23 | HanXRQChr04g0112331 | 2 | 3 |
|  | HanPP2C29 | HanXRQChr05g0144811 | 3 | 4 |
|  | HanPP2C48 | HanXRQChr08g0216371 | 3 | 4 |
|  | HanPP2C52 | HanXRQChr08g0236301 | 3 | 4 |
|  | HanPP2C59 | HanXRQChr09g0243021 | 3 | 4 |
|  | HanPP2C66 | HanXRQChr09g0259441 | 3 | 4 |
|  | HanPP2C89 | HanXRQChr12g0367111 | 2 | 3 |
|  | HanPP2C97 | HanXRQChr13g0413411 | 4 | 5 |
|  | HanPP2C107 | HanXRQChr15g0493551 | 3 | 4 |
|  | HanPP2C112 | HanXRQChr16g0512471 | 3 | 4 |
| H | HanPP2C3 | HanXRQChr01g0011811 | 6 | 7 |
|  | HanPP2C11 | HanXRQChr02g0037691 | 8 | 9 |
|  | HanPP2C12 | HanXRQChr02g0050381 | 2 | 3 |
|  | HanPP2C33 | HanXRQChr06g0177471 | 2 | 3 |
|  | HanPP2C67 | HanXRQChr09g0267901 | 7 | 8 |
|  | HanPP2C78 | HanXRQChr10g0303991 | 8 | 9 |
|  | HanPP2C115 | HanXRQChr16g0529151 | 7 | 8 |
|  | HanPP2C120 | HanXRQChr17g0551841 | 7 | 8 |
| I | HanPP2C10 | HanXRQChr02g0034061 | 9 | 10 |
|  | HanPP2C19 | HanXRQChr03g0080041 | 9 | 10 |
|  | HanPP2C31 | HanXRQChr05g0153691 | 9 | 10 |
|  | HanPP2C95 | HanXRQChr13g0411021 | 9 | 10 |
| J | HanPP2C55 | HanXRQChr09g0240611 | 1 | 2 |
|  | HanPP2C56 | HanXRQChr09g0240651 | 1 | 2 |
|  | HanPP2C57 | HanXRQChr09g0240681 | 0 | 1 |
|  | HanPP2C58 | HanXRQChr09g0240711 | 1 | 2 |
|  | HanPP2C70 | HanXRQChr10g0281961 | 19 | 20 |
|  | HanPP2C84 | HanXRQChr11g0338821 | 5 | 6 |
| L | HanPP2C38 | HanXRQChr07g0192671 | 2 | 3 |
|  | HanPP2C109 | HanXRQChr16g0507681 | 14 | 15 |
| Outgroup | HanPP2C22 | HanXRQChr04g0094871 | 5 | 6 |
|  | HanPP2C37 | HanXRQChr07g0192431 | 3 | 4 |
|  | HanPP2C45 | HanXRQChr07g0206421 | 9 | 10 |

**S6 Data** (Continued)

| **Group** | **PP2C gene** | **Source accession** | **Intron** | **Exon** |
| --- | --- | --- | --- | --- |
|  | HanPP2C49 | HanXRQChr08g0221041 | 0 | 1 |
|  | HanPP2C76 | HanXRQChr10g0298501 | 11 | 12 |
|  | HanPP2C93 | HanXRQChr13g0400271 | 12 | 13 |
|  | HanPP2C106 | HanXRQChr15g0493161 | 2 | 3 |
