# Supplementary material for: Genome-wide identification and characterization of protein phosphatase 2C (PP2C) gene family in sunflower (Helianthus annuus L.) and their expression profiles in response to multiple abiotic stresses
Source: PLoS One. 2024 Mar 20;19(3):e0298543. doi: 10.1371/journal.pone.0298543 (PMC10954154; doi:10.1371/journal.pone.0298543)
Supplement: S10 Data — The miRNA data was downloaded from the plant micro-RNA encyclopedia (http://pmiren.com/). (DOCX) [file pone.0298543.s010.docx]

**S10 Data. miRNA targeted prediction of HanPP2C. The miRNA data was downloaded from the plant *micro* RNA encyclopedia (**[**http://pmiren.com/**](http://pmiren.com/)**).**

| **miRNA ID** | **Target ID** | **Target length** | **Target**  **start** | **Target**  **end** | **miRNA_aligned_fragment** |
| --- | --- | --- | --- | --- | --- |
| Han-miR156b | HanPP2C100 | 21 | 1233 | 1252 | UUGACAGAAGAGAGUGAGCAC |
| Han-miR156c | HanPP2C100 | 21 | 1233 | 1252 | UUGACAGAAGAGAGUGAGCAC |
| Han-miR170b | HanPP2C19 | 21 | 882 | 902 | UUGAGCCGCGCCAAUAUCACU |
| Han-miR170b | HanPP2C95 | 21 | 885 | 905 | UUGAGCCGCGCCAAUAUCACU |
| Han-miR170e | HanPP2C95 | 21 | 885 | 905 | UUGAGCCGCGCCAAUAUCACU |
| Han-miR170e | HanPP2C19 | 21 | 882 | 902 | UUGAGCCGCGCCAAUAUCACU |
| Han-miRN5691 | HanPP2C45 | 22 | 560 | 580 | UUCCAGAUUGUGUAGAGAACGC |
| Han-miRN5691 | HanPP2C37 | 22 | 221 | 241 | UUCCAGAUUGUGUAGAGAACGC |
| Han-miRN5704 | HanPP2C11 | 20 | 311 | 330 | CAGAGCCUUAGGCAGAACGG |
| Han-miRN5706 | HanPP2C11 | 20 | 311 | 330 | CAGAGCCUUAGGCAGAACGG |
| Han-miR156b | HanPP2C70 | 21 | 422 | 442 | UUGACAGAAGAGAGUGAGCAC |
| Han-miR156c | HanPP2C70 | 21 | 422 | 442 | UUGACAGAAGAGAGUGAGCAC |
| Han-miR170a | HanPP2C10 | 21 | 965 | 985 | UGAGCCGAACCAGUAUCACUC |
| Han-miR171a | HanPP2C95 | 22 | 884 | 905 | UUGAGCCGUGCCAAUAUCACGU |
| Han-miR5675c | HanPP2C51 | 21 | 171 | 191 | UUGGAGACUGAUCGGAAUUGA |
| Han-miRN5685 | HanPP2C38 | 22 | 491 | 512 | UUUGGAUCAUCCGCUUUGGGGC |
| Han-miRN5691 | HanPP2C17 | 22 | 676 | 697 | UUCCAGAUUGUGUAGAGAACGC |
| Han-miRN5715 | HanPP2C33 | 21 | 249 | 269 | UAAACAUUGUUCAACAGGACA |
| Han-miRN5715 | HanPP2C11 | 21 | 249 | 269 | UAAACAUUGUUCAACAGGACA |
| Han-miRN5747 | HanPP2C114 | 21 | 1057 | 1077 | UUGGGGGAAAGUAUCAUCAUC |
| Han-miRN5763 | HanPP2C22 | 20 | 708 | 727 | GUCGGGAUAGCUCAGUUGGA |
| Han-miRN5764 | HanPP2C11 | 21 | 249 | 269 | UAAACAUUGUUCAACAGGACA |
| Han-miRN5764 | HanPP2C33 | 21 | 249 | 269 | UAAACAUUGUUCAACAGGACA |
| Han-miRN5772 | HanPP2C11 | 21 | 249 | 269 | UAAACAUUGUUCAACAGGACA |
| Han-miRN5772 | HanPP2C33 | 21 | 249 | 269 | UAAACAUUGUUCAACAGGACA |
| Han-miRN5773 | HanPP2C33 | 21 | 249 | 269 | UAAACAUUGUUCAACAGGACA |
| Han-miRN5773 | HanPP2C11 | 21 | 249 | 269 | UAAACAUUGUUCAACAGGACA |
| Han-miR1516a | HanPP2C88 | 22 | 513 | 534 | UUUGUUCUCAAUAGCCUUCCAA |
| Han-miR1516b | HanPP2C88 | 22 | 513 | 534 | UUUGUUCUCAAUAGCCUUCCAA |
| Han-miR156a | HanPP2C70 | 21 | 421 | 441 | UGACAGAAGAGAGUGAGCACU |
| Han-miR156d | HanPP2C70 | 21 | 421 | 441 | UGACAGAAGAGAGUGAGCAUA |
| Han-miR156h | HanPP2C70 | 21 | 421 | 441 | UGACAGAAGAGAGUGAGCAUA |
| Han-miR156i | HanPP2C100 | 21 | 1233 | 1252 | CUGACAGAAGAGAGUGAGCAC |
| Han-miR167a | HanPP2C84 | 21 | 740 | 760 | UGAAGCUGCCAGCAUGAUCUA |
| Han-miR167a | HanPP2C61 | 21 | 521 | 541 | UGAAGCUGCCAGCAUGAUCUA |
| Han-miR167a | HanPP2C72 | 21 | 521 | 541 | UGAAGCUGCCAGCAUGAUCUA |
| Han-miR167b | HanPP2C84 | 21 | 740 | 760 | UGAAGCUGCCAGCAUGAUCUA |
| Han-miR167b | HanPP2C61 | 21 | 521 | 541 | UGAAGCUGCCAGCAUGAUCUA |
| Han-miR167b | HanPP2C72 | 21 | 521 | 541 | UGAAGCUGCCAGCAUGAUCUA |

**S10 Data** (Continued)

| **miRNA ID** | **Target ID** | **Target length** | **Target**  **start** | **Target**  **end** | **miRNA_aligned_fragment** |
| --- | --- | --- | --- | --- | --- |
| Han-miR167c | HanPP2C84 | 21 | 740 | 760 | UGAAGCUGCCAGCAUGAUCUA |
| Han-miR167c | HanPP2C61 | 21 | 521 | 541 | UGAAGCUGCCAGCAUGAUCUA |
| Han-miR167c | HanPP2C72 | 21 | 521 | 541 | UGAAGCUGCCAGCAUGAUCUA |
| Han-miR167d | HanPP2C84 | 21 | 740 | 760 | UGAAGCUGCCAGCAUGAUCUA |
| Han-miR167d | HanPP2C61 | 21 | 521 | 541 | UGAAGCUGCCAGCAUGAUCUA |
| Han-miR167d | HanPP2C72 | 21 | 521 | 541 | UGAAGCUGCCAGCAUGAUCUA |
| Han-miR167e | HanPP2C84 | 21 | 740 | 760 | UGAAGCUGCCAGCAUGAUCUG |
| Han-miR167e | HanPP2C61 | 21 | 521 | 541 | UGAAGCUGCCAGCAUGAUCUG |
| Han-miR169a | HanPP2C26 | 21 | 298 | 318 | GAGCCAAGGAUGAAUUGCCGG |
| Han-miR169c | HanPP2C79 | 20 | 603 | 622 | GGCAGUCUUCUUGGCUAACC |
| Han-miR169m | HanPP2C79 | 20 | 603 | 622 | GGCAGUCUUCUUGGCUAACC |
| Han-miR393a | HanPP2C119 | 22 | 498 | 519 | UCCAAAGGGAUCGCAUUGAUCC |
| Han-miR393b | HanPP2C119 | 22 | 498 | 519 | UCCAAAGGGAUCGCAUUGAUCC |
| Han-miR393c | HanPP2C119 | 22 | 498 | 519 | UCCAAAGGGAUCGCAUUGAUCC |
| Han-miR393d | HanPP2C119 | 22 | 498 | 519 | UCCAAAGGGAUCGCAUUGAUCC |
| Han-miR393e | HanPP2C119 | 22 | 498 | 519 | UCCAAAGGGAUCGCAUUGAUCC |
| Han-miR394a | HanPP2C22 | 20 | 639 | 658 | UUGGCAUUCUGUCCACCUCC |
| Han-miR394b | HanPP2C22 | 20 | 639 | 658 | UUGGCAUUCUGUCCACCUCC |
| Han-miR397a | HanPP2C81 | 21 | 1906 | 1926 | UCAUUGAGUGCAGCGUUGAUG |
| Han-miR408a | HanPP2C49 | 21 | 165 | 185 | UGCACUGUCUCUUCCCUGGCU |
| Han-miR5675b | HanPP2C43 | 22 | 819 | 840 | UUCCGAGACCACCUAUUCCAAC |
| Han-miR5675b | HanPP2C44 | 22 | 819 | 840 | UUCCGAGACCACCUAUUCCAAC |
| Han-miR5675b | HanPP2C42 | 22 | 819 | 840 | UUCCGAGACCACCUAUUCCAAC |
| Han-miR5675c | HanPP2C25 | 21 | 183 | 203 | UUGGAGACUGAUCGGAAUUGA |
| Han-miR858a | HanPP2C36 | 21 | 645 | 665 | UUCGUUGUCUGUUCGACCUUG |
| Han-miRN1650a | HanPP2C98 | 22 | 795 | 816 | UUAAACAGAGAAAUCACGAUUG |
| Han-miRN5680 | HanPP2C30 | 21 | 580 | 600 | CAUGUGCCCAUCUUCCCCAUC |
| Han-miRN5699 | HanPP2C69 | 21 | 631 | 651 | UAAAACACUUAGGUUGGGAAG |
| Han-miRN5700 | HanPP2C109 | 21 | 1786 | 1806 | UGAGAUGGACGGCUUAGAGUC |
| Han-miRN5703 | HanPP2C58 | 21 | 999 | 1019 | AAUAAAUGUAAAGGACUGCCU |
| Han-miRN5704 | HanPP2C33 | 20 | 311 | 330 | CAGAGCCUUAGGCAGAACGG |
| Han-miRN5705 | HanPP2C84 | 22 | 594 | 615 | AAGAAUAACAAUGGCGGAUCAU |
| Han-miRN5706 | HanPP2C33 | 20 | 311 | 330 | CAGAGCCUUAGGCAGAACGG |
| Han-miRN5713 | HanPP2C30 | 21 | 580 | 600 | CAUGUGCCCAUCUUCCCCAUC |
| Han-miRN5738 | HanPP2C2 | 21 | 513 | 533 | UUCUUUUAGGGCUUUUCCGAC |
| Han-miRN5738 | HanPP2C70 | 21 | 2539 | 2559 | UUCUUUUAGGGCUUUUCCGAC |
| Han-miRN5738 | HanPP2C24 | 21 | 77 | 97 | UUCUUUUAGGGCUUUUCCGAC |
| Han-miRN5746 | HanPP2C20 | 21 | 962 | 982 | UAGAAUAGGUGGCUUGGAACA |
| Han-miRN5746 | HanPP2C96 | 21 | 695 | 715 | UAGAAUAGGUGGCUUGGAACA |
| Han-miRN5747 | HanPP2C48 | 21 | 1041 | 1060 | UUGGGGGAAAGUAUCAUCAUC |

**S10 Data** (Continued)

| **miRNA ID** | **Target ID** | **Target length** | **Target**  **start** | **Target**  **end** | **miRNA_aligned_fragment** |
| --- | --- | --- | --- | --- | --- |
| Han-miRN5761 | HanPP2C33 | 21 | 249 | 269 | UAGACAUUGUUCAACAGGACA |
| Han-miRN5761 | HanPP2C11 | 21 | 249 | 269 | UAGACAUUGUUCAACAGGACA |
| Han-miRN5768 | HanPP2C66 | 21 | 161 | 181 | UUAGAGACGGAUCUGAAUUGG |
| Han-miR1516a | HanPP2C10 | 22 | 681 | 702 | UUUGUUCUCAAUAGCCUUCCAA |
| Han-miR1516a | HanPP2C81 | 22 | 2081 | 2102 | UUUGUUCUCAAUAGCCUUCCAA |
| Han-miR1516a | HanPP2C81 | 22 | 252 | 273 | UUUGUUCUCAAUAGCCUUCCAA |
| Han-miR1516a | HanPP2C103 | 22 | 923 | 944 | UUUGUUCUCAAUAGCCUUCCAA |
| Han-miR1516b | HanPP2C10 | 22 | 681 | 702 | UUUGUUCUCAAUAGCCUUCCAA |
| Han-miR1516b | HanPP2C81 | 22 | 2081 | 2102 | UUUGUUCUCAAUAGCCUUCCAA |
| Han-miR1516b | HanPP2C81 | 22 | 252 | 273 | UUUGUUCUCAAUAGCCUUCCAA |
| Han-miR1516b | HanPP2C103 | 22 | 923 | 944 | UUUGUUCUCAAUAGCCUUCCAA |
| Han-miR1516c | HanPP2C21 | 22 | 483 | 504 | AUGAAGCUUAAAAACACAUCGU |
| Han-miR1516c | HanPP2C98 | 22 | 474 | 495 | AUGAAGCUUAAAAACACAUCGU |
| Han-miR156a | HanPP2C114 | 21 | 1093 | 1113 | UGACAGAAGAGAGUGAGCACU |
| Han-miR156b | HanPP2C114 | 21 | 1094 | 1114 | UUGACAGAAGAGAGUGAGCAC |
| Han-miR156c | HanPP2C114 | 21 | 1094 | 1114 | UUGACAGAAGAGAGUGAGCAC |
| Han-miR156e | HanPP2C14 | 21 | 495 | 515 | UUGACAGAAGAUAGAGAGCAC |
| Han-miR156f | HanPP2C14 | 21 | 495 | 515 | UUGACAGAAGAUAGAGAGCAC |
| Han-miR156g | HanPP2C14 | 21 | 495 | 515 | UUGACAGAAGAUAGAGAGCAC |
| Han-miR156i | HanPP2C70 | 21 | 422 | 442 | CUGACAGAAGAGAGUGAGCAC |
| Han-miR156j | HanPP2C14 | 21 | 495 | 515 | UUGACAGAAGAUAGAGAGCAC |
| Han-miR156k | HanPP2C14 | 21 | 495 | 515 | UUGACAGAAGAUAGAGAGCAC |
| Han-miR156l | HanPP2C14 | 21 | 495 | 515 | UUGACAGAAGAUAGAGAGCAC |
| Han-miR156m | HanPP2C14 | 21 | 495 | 515 | UUGACAGAAGAUAGAGAGCAC |
| Han-miR159b | HanPP2C109 | 20 | 975 | 994 | CUUGGAUUGAAGGGAGCUCU |
| Han-miR159b | HanPP2C93 | 20 | 769 | 788 | CUUGGAUUGAAGGGAGCUCU |
| Han-miR159g | HanPP2C109 | 20 | 975 | 994 | CUUGGAUUGAAGGGAGCUCU |
| Han-miR159g | HanPP2C93 | 20 | 769 | 788 | CUUGGAUUGAAGGGAGCUCU |
| Han-miR159h | HanPP2C109 | 20 | 975 | 994 | CUUGGAUUGAAGGGAGCUCU |
| Han-miR159h | HanPP2C93 | 20 | 769 | 788 | CUUGGAUUGAAGGGAGCUCU |
| Han-miR162a | HanPP2C47 | 21 | 316 | 336 | UCGAUAAACCUCUGCAUCCAG |
| Han-miR162b | HanPP2C47 | 21 | 316 | 336 | UCGAUAAACCUCUGCAUCCAG |
| Han-miR167a | HanPP2C15 | 21 | 734 | 754 | UGAAGCUGCCAGCAUGAUCUA |
| Han-miR167b | HanPP2C15 | 21 | 734 | 754 | UGAAGCUGCCAGCAUGAUCUA |
| Han-miR167c | HanPP2C15 | 21 | 734 | 754 | UGAAGCUGCCAGCAUGAUCUA |
| Han-miR167d | HanPP2C15 | 21 | 734 | 754 | UGAAGCUGCCAGCAUGAUCUA |
| Han-miR167e | HanPP2C15 | 21 | 734 | 754 | UGAAGCUGCCAGCAUGAUCUG |
| Han-miR168a | HanPP2C29 | 21 | 1072 | 1092 | UCGCUUGGUACAGGUCGGGAA |
| Han-miR168b | HanPP2C29 | 21 | 1072 | 1092 | UCGCUUGGUACAGGUCGGGAA |
| Han-miR169c | HanPP2C75 | 20 | 350 | 369 | GGCAGUCUUCUUGGCUAACC |

**S10 Data** (Continued)

| **miRNA ID** | **Target ID** | **Target length** | **Target**  **start** | **Target**  **end** | **miRNA_aligned_fragment** |
| --- | --- | --- | --- | --- | --- |
| Han-miR169m | HanPP2C75 | 20 | 350 | 369 | GGCAGUCUUCUUGGCUAACC |
| Han-miR169t | HanPP2C97 | 21 | 386 | 406 | CAGCCAAGGAUGACUUGCCGC |
| Han-miR170b | HanPP2C111 | 21 | 518 | 538 | UUGAGCCGCGCCAAUAUCACU |
| Han-miR170c | HanPP2C32 | 21 | 86 | 106 | UGAUUGAGUCGUGCCAAUAUC |
| Han-miR170e | HanPP2C111 | 21 | 518 | 538 | UUGAGCCGCGCCAAUAUCACU |
| Han-miR170f | HanPP2C78 | 21 | 549 | 568 | UGAGCCGAACCAAUAUCACUC |
| Han-miR170f | HanPP2C67 | 21 | 555 | 574 | UGAGCCGAACCAAUAUCACUC |
| Han-miR170g | HanPP2C78 | 21 | 549 | 568 | UGAGCCGAACCAAUAUCACUC |
| Han-miR170g | HanPP2C67 | 21 | 555 | 574 | UGAGCCGAACCAAUAUCACUC |
| Han-miR170h | HanPP2C78 | 21 | 549 | 568 | UGAGCCGAACCAAUAUCACUC |
| Han-miR170h | HanPP2C67 | 21 | 555 | 574 | UGAGCCGAACCAAUAUCACUC |
| Han-miR171a | HanPP2C26 | 22 | 739 | 760 | UUGAGCCGUGCCAAUAUCACGU |
| Han-miR172a | HanPP2C67 | 21 | 867 | 887 | AGAAUCUUGAUGAUGCUGCAU |
| Han-miR172a | HanPP2C64 | 21 | 1006 | 1026 | AGAAUCUUGAUGAUGCUGCAU |
| Han-miR172a | HanPP2C22 | 21 | 285 | 305 | AGAAUCUUGAUGAUGCUGCAU |
| Han-miR172b | HanPP2C67 | 21 | 867 | 887 | AGAAUCUUGAUGAUGCUGCAU |
| Han-miR172b | HanPP2C64 | 21 | 1006 | 1026 | AGAAUCUUGAUGAUGCUGCAU |
| Han-miR172b | HanPP2C22 | 21 | 285 | 305 | AGAAUCUUGAUGAUGCUGCAU |
| Han-miR172c | HanPP2C67 | 21 | 867 | 887 | AGAAUCUUGAUGAUGCUGCAU |
| Han-miR172c | HanPP2C64 | 21 | 1006 | 1026 | AGAAUCUUGAUGAUGCUGCAU |
| Han-miR172c | HanPP2C22 | 21 | 285 | 305 | AGAAUCUUGAUGAUGCUGCAU |
| Han-miR172d | HanPP2C67 | 21 | 867 | 887 | AGAAUCUUGAUGAUGCUGCAU |
| Han-miR172d | HanPP2C64 | 21 | 1006 | 1026 | AGAAUCUUGAUGAUGCUGCAU |
| Han-miR172d | HanPP2C22 | 21 | 285 | 305 | AGAAUCUUGAUGAUGCUGCAU |
| Han-miR172e | HanPP2C64 | 21 | 1006 | 1026 | AGAAUCUUGAUGAUGCUGCAU |
| Han-miR172e | HanPP2C67 | 21 | 867 | 887 | AGAAUCUUGAUGAUGCUGCAU |
| Han-miR172e | HanPP2C22 | 21 | 285 | 305 | AGAAUCUUGAUGAUGCUGCAU |
| Han-miR172f | HanPP2C64 | 21 | 1006 | 1026 | AGAAUCUUGAUGAUGCUGCAU |
| Han-miR172f | HanPP2C67 | 21 | 867 | 887 | AGAAUCUUGAUGAUGCUGCAU |
| Han-miR172f | HanPP2C22 | 21 | 285 | 305 | AGAAUCUUGAUGAUGCUGCAU |
| Han-miR172g | HanPP2C64 | 21 | 1006 | 1026 | CGAAUCUUGAUGAUGCUGCAG |
| Han-miR172g | HanPP2C118 | 21 | 317 | 337 | CGAAUCUUGAUGAUGCUGCAG |
| Han-miR172g | HanPP2C71 | 21 | 260 | 280 | CGAAUCUUGAUGAUGCUGCAG |
| Han-miR172h | HanPP2C22 | 21 | 286 | 306 | GAGAAUCUUGAUGAUGCUGCA |
| Han-miR172h | HanPP2C38 | 21 | 316 | 336 | GAGAAUCUUGAUGAUGCUGCA |
| Han-miR172i | HanPP2C22 | 21 | 286 | 306 | GAGAAUCUUGAUGAUGCUGCA |
| Han-miR172i | HanPP2C38 | 21 | 316 | 336 | GAGAAUCUUGAUGAUGCUGCA |
| Han-miR172j | HanPP2C22 | 21 | 286 | 306 | GAGAAUCUUGAUGAUGCUGCA |
| Han-miR172j | HanPP2C38 | 21 | 316 | 336 | GAGAAUCUUGAUGAUGCUGCA |
| Han-miR390a | HanPP2C33 | 21 | 271 | 291 | AAGCUCAGGAGGGAUAACGCC |

**S10 Data** (Continued)

| **miRNA ID** | **Target ID** | **Target length** | **Target**  **start** | **Target**  **end** | **miRNA_aligned_fragment** |
| --- | --- | --- | --- | --- | --- |
| Han-miR390b | HanPP2C11 | 21 | 271 | 291 | AAGCUCAGGAGGGAUAGCGCC |
| Han-miR390b | HanPP2C78 | 21 | 268 | 288 | AAGCUCAGGAGGGAUAGCGCC |
| Han-miR390c | HanPP2C11 | 21 | 271 | 291 | AAGCUCAGGAGGGAUAGCGCC |
| Han-miR390c | HanPP2C78 | 21 | 268 | 288 | AAGCUCAGGAGGGAUAGCGCC |
| Han-miR390d | HanPP2C11 | 21 | 271 | 291 | AAGCUCAGGAGGGAUAGCGCC |
| Han-miR390d | HanPP2C78 | 21 | 268 | 288 | AAGCUCAGGAGGGAUAGCGCC |
| Han-miR393a | HanPP2C72 | 22 | 178 | 198 | UCCAAAGGGAUCGCAUUGAUCC |
| Han-miR393b | HanPP2C72 | 22 | 178 | 198 | UCCAAAGGGAUCGCAUUGAUCC |
| Han-miR393c | HanPP2C72 | 22 | 178 | 198 | UCCAAAGGGAUCGCAUUGAUCC |
| Han-miR393d | HanPP2C72 | 22 | 178 | 198 | UCCAAAGGGAUCGCAUUGAUCC |
| Han-miR393e | HanPP2C72 | 22 | 178 | 198 | UCCAAAGGGAUCGCAUUGAUCC |
| Han-miR395a | HanPP2C38 | 21 | 1147 | 1167 | CUGAAGAGUUUGGGGGAACUU |
| Han-miR396a | HanPP2C27 | 21 | 581 | 601 | UUCCACAGCUUUCUUGAACUG |
| Han-miR396b | HanPP2C27 | 21 | 581 | 601 | UUCCACAGCUUUCUUGAACUG |
| Han-miR396c | HanPP2C107 | 21 | 481 | 501 | UUCCACGGCUUUCUUGAACUG |
| Han-miR396c | HanPP2C11 | 21 | 1012 | 1032 | UUCCACGGCUUUCUUGAACUG |
| Han-miR396d | HanPP2C27 | 21 | 581 | 601 | UUCCACAGCUUUCUUGAACUG |
| Han-miR396e | HanPP2C27 | 21 | 581 | 601 | UUCCACAGCUUUCUUGAACUG |
| Han-miR396f | HanPP2C107 | 21 | 481 | 501 | UUCCACGGCUUUCUUGAACUG |
| Han-miR396f | HanPP2C11 | 21 | 1012 | 1032 | UUCCACGGCUUUCUUGAACUG |
| Han-miR396g | HanPP2C27 | 21 | 581 | 601 | UUCCACAGCUUUCUUGAACUU |
| Han-miR396h | HanPP2C27 | 21 | 581 | 601 | UUCCACAGCUUUCUUGAACUU |
| Han-miR397a | HanPP2C46 | 21 | 411 | 430 | UCAUUGAGUGCAGCGUUGAUG |
| Han-miR397a | HanPP2C60 | 21 | 951 | 971 | UCAUUGAGUGCAGCGUUGAUG |
| Han-miR397a | HanPP2C40 | 21 | 327 | 347 | UCAUUGAGUGCAGCGUUGAUG |
| Han-miR397a | HanPP2C39 | 21 | 327 | 347 | UCAUUGAGUGCAGCGUUGAUG |
| Han-miR397a | HanPP2C4 | 21 | 288 | 308 | UCAUUGAGUGCAGCGUUGAUG |
| Han-miR403a | HanPP2C60 | 21 | 302 | 322 | UUAGAUUCACGCACAAACUCG |
| Han-miR482a | HanPP2C108 | 22 | 421 | 442 | UUACCUACUCCACCCAUGCCAA |
| Han-miR482a | HanPP2C106 | 22 | 547 | 568 | UUACCUACUCCACCCAUGCCAA |
| Han-miR530a | HanPP2C79 | 22 | 477 | 498 | UGCAUUUGCACCUGCACCUUUC |
| Han-miR530a | HanPP2C25 | 22 | 1108 | 1129 | UGCAUUUGCACCUGCACCUUUC |
| Han-miR530b | HanPP2C79 | 21 | 478 | 498 | UGCAUUUGCACCUGCACCUUC |
| Han-miR530b | HanPP2C25 | 21 | 1109 | 1129 | UGCAUUUGCACCUGCACCUUC |
| Han-miR5675a | HanPP2C23 | 21 | 424 | 444 | GCUCACCCUCUAUCUGUCACC |
| Han-miR5675a | HanPP2C76 | 21 | 1630 | 1650 | GCUCACCCUCUAUCUGUCACC |
| Han-miR5675b | HanPP2C102 | 22 | 858 | 879 | UUCCGAGACCACCUAUUCCAAC |
| Han-miR5675c | HanPP2C81 | 21 | 180 | 200 | UUGGAGACUGAUCGGAAUUGA |
| Han-miR5675c | HanPP2C84 | 21 | 122 | 142 | UUGGAGACUGAUCGGAAUUGA |
| Han-miR6113a | HanPP2C80 | 22 | 356 | 377 | UCUGAAACUCAAGAACACGUCG |

**S10 Data** (Continued)

| **miRNA ID** | **Target ID** | **Target length** | **Target**  **start** | **Target**  **end** | **miRNA_aligned_fragment** |
| --- | --- | --- | --- | --- | --- |
| Han-miR7122a | HanPP2C21 | 22 | 804 | 825 | UUAAACAGAGAAAUCACGGUUG |
| Han-miR7122b | HanPP2C21 | 22 | 804 | 825 | UUAAACAGAGAAAUCACGGUUG |
| Han-miR858a | HanPP2C40 | 21 | 829 | 849 | UUCGUUGUCUGUUCGACCUUG |
| Han-miR858a | HanPP2C119 | 21 | 763 | 783 | UUCGUUGUCUGUUCGACCUUG |
| Han-miR858a | HanPP2C41 | 21 | 787 | 807 | UUCGUUGUCUGUUCGACCUUG |
| Han-miRN17a | HanPP2C87 | 21 | 880 | 900 | CGCGGCGUCGUCAUUGCACCG |
| Han-miRN1a | HanPP2C115 | 22 | 550 | 571 | UGCUCACUUCUCUUCUGUCAGC |
| Han-miRN1b | HanPP2C115 | 22 | 550 | 571 | UGCUCACUUCUCUUCUGUCAGC |
| Han-miRN5683 | HanPP2C89 | 21 | 16 | 36 | UGUACAAGAGCCCGAACGCCU |
| Han-miRN5685 | HanPP2C69 | 22 | 260 | 281 | UUUGGAUCAUCCGCUUUGGGGC |
| Han-miRN5689 | HanPP2C105 | 21 | 911 | 931 | AUCGAGCUGCACAAUACACUG |
| Han-miRN5690 | HanPP2C105 | 21 | 911 | 931 | AUCGAGCUGCACAAUACACUG |
| Han-miRN5692 | HanPP2C105 | 21 | 911 | 931 | AUCGAGCUGCACAAUACACUG |
| Han-miRN5696 | HanPP2C23 | 21 | 424 | 444 | GCUCACCCUCUAUCUGUCACC |
| Han-miRN5696 | HanPP2C76 | 21 | 1630 | 1650 | GCUCACCCUCUAUCUGUCACC |
| Han-miRN5697 | HanPP2C23 | 21 | 424 | 444 | GCUCACCCUCUAUCUGUCACC |
| Han-miRN5697 | HanPP2C76 | 21 | 1630 | 1650 | GCUCACCCUCUAUCUGUCACC |
| Han-miRN5698 | HanPP2C22 | 22 | 895 | 916 | UAAGGUGAACCAAAGAACAAUU |
| Han-miRN5699 | HanPP2C5 | 21 | 34 | 54 | UAAAACACUUAGGUUGGGAAG |
| Han-miRN5700 | HanPP2C120 | 21 | 935 | 955 | UGAGAUGGACGGCUUAGAGUC |
| Han-miRN5703 | HanPP2C70 | 21 | 1587 | 1607 | AAUAAAUGUAAAGGACUGCCU |
| Han-miRN5704 | HanPP2C78 | 20 | 308 | 327 | CAGAGCCUUAGGCAGAACGG |
| Han-miRN5704 | HanPP2C67 | 20 | 314 | 333 | CAGAGCCUUAGGCAGAACGG |
| Han-miRN5705 | HanPP2C52 | 22 | 960 | 981 | AAGAAUAACAAUGGCGGAUCAU |
| Han-miRN5705 | HanPP2C55 | 22 | 228 | 249 | AAGAAUAACAAUGGCGGAUCAU |
| Han-miRN5706 | HanPP2C78 | 20 | 308 | 327 | CAGAGCCUUAGGCAGAACGG |
| Han-miRN5706 | HanPP2C67 | 20 | 314 | 333 | CAGAGCCUUAGGCAGAACGG |
| Han-miRN5708 | HanPP2C67 | 20 | 314 | 333 | CAGAGCCUUGAGCAGAACGA |
| Han-miRN5709 | HanPP2C81 | 21 | 446 | 466 | GGAAGCCUGAGAGGUAGGACG |
| Han-miRN5709 | HanPP2C14 | 21 | 88 | 108 | GGAAGCCUGAGAGGUAGGACG |
| Han-miRN5710 | HanPP2C12 | 21 | 142 | 162 | CGAUUGACUCUCCUUGCCACC |
| Han-miRN5711 | HanPP2C95 | 21 | 101 | 121 | CACAGGACUUAUCGAACACCC |
| Han-miRN5715 | HanPP2C25 | 21 | 925 | 945 | UAAACAUUGUUCAACAGGACA |
| Han-miRN5715 | HanPP2C49 | 21 | 343 | 363 | UAAACAUUGUUCAACAGGACA |
| Han-miRN5720 | HanPP2C74 | 21 | 226 | 246 | UGGAGACGGAUCUGAAUUGGA |
| Han-miRN5720 | HanPP2C66 | 21 | 160 | 180 | UGGAGACGGAUCUGAAUUGGA |
| Han-miRN5724 | HanPP2C34 | 22 | 1031 | 1053 | CUUCAUCUCUGGAUA-GGUCUUC |
| Han-miRN5724 | HanPP2C70 | 22 | 2657 | 2677 | CUUCAUCUCUGGAUAGGUCUUC |
| Han-miRN5728 | HanPP2C84 | 21 | 892 | 912 | UGAGAGAACCAAGGAUCCAUC |
| Han-miRN5730 | HanPP2C94 | 21 | 758 | 778 | UUGCGAUUUUUGUUUGUGCUU |

**S10 Data** (Continued)

| **miRNA ID** | **Target ID** | **Target length** | **Target**  **start** | **Target**  **end** | **miRNA_aligned_fragment** |
| --- | --- | --- | --- | --- | --- |
| Han-miRN5738 | HanPP2C102 | 21 | 239 | 259 | UUCUUUUAGGGCUUUUCCGAC |
| Han-miRN5739 | HanPP2C65 | 21 | 513 | 532 | GCUCACCCUCUGUCUGUCACA |
| Han-miRN5741 | HanPP2C108 | 22 | 421 | 442 | UUGCCUACCCCACCCAUGCCAA |
| Han-miRN5744 | HanPP2C25 | 22 | 1070 | 1090 | UCUUCCCCACACCGCCCAUUCC |
| Han-miRN5744 | HanPP2C86 | 22 | 504 | 525 | UCUUCCCCACACCGCCCAUUCC |
| Han-miRN5744 | HanPP2C113 | 22 | 685 | 706 | UCUUCCCCACACCGCCCAUUCC |
| Han-miRN5747 | HanPP2C46 | 21 | 1065 | 1085 | UUGGGGGAAAGUAUCAUCAUC |
| Han-miRN5748 | HanPP2C3 | 21 | 56 | 76 | GCUCAUGAUCUAUCUGUCAGC |
| Han-miRN5750 | HanPP2C3 | 21 | 56 | 76 | GCUCAUGAUCUAUCUGUCAGC |
| Han-miRN5751 | HanPP2C3 | 21 | 56 | 76 | GCUCAUGAUCUAUCUGUCAGC |
| Han-miRN5754 | HanPP2C9 | 21 | 540 | 561 | GCUC-ACUCUCUAUCUGUCAUC |
| Han-miRN5754 | HanPP2C23 | 21 | 424 | 444 | GCUCACUCUCUAUCUGUCAUC |
| Han-miRN5755 | HanPP2C9 | 21 | 540 | 561 | GCUC-ACUCUCUAUCUGUCAUC |
| Han-miRN5755 | HanPP2C23 | 21 | 424 | 444 | GCUCACUCUCUAUCUGUCAUC |
| Han-miRN5763 | HanPP2C70 | 20 | 1335 | 1354 | GUCGGGAUAGCUCAGUUGGA |
| Han-miRN5764 | HanPP2C25 | 21 | 925 | 945 | UAAACAUUGUUCAACAGGACA |
| Han-miRN5764 | HanPP2C49 | 21 | 343 | 363 | UAAACAUUGUUCAACAGGACA |
| Han-miRN5768 | HanPP2C65 | 21 | 200 | 220 | UUAGAGACGGAUCUGAAUUGG |
| Han-miRN5772 | HanPP2C25 | 21 | 925 | 945 | UAAACAUUGUUCAACAGGACA |
| Han-miRN5772 | HanPP2C49 | 21 | 343 | 363 | UAAACAUUGUUCAACAGGACA |
| Han-miRN5773 | HanPP2C25 | 21 | 925 | 945 | UAAACAUUGUUCAACAGGACA |
| Han-miRN5773 | HanPP2C49 | 21 | 343 | 363 | UAAACAUUGUUCAACAGGACA |
| Han-miRN5774 | HanPP2C38 | 22 | 500 | 521 | UUUGCAGUUUUCCAGUCACAUG |
