# Supplementary figures and images for: Genome-wide identification and characterization of protein phosphatase 2C (PP2C) gene family in sunflower (Helianthus annuus L.) and their expression profiles in response to multiple abiotic stresses
Source: PLoS One. 2024 Mar 20;19(3):e0298543. doi: 10.1371/journal.pone.0298543 (PMC10954154; doi:10.1371/journal.pone.0298543)

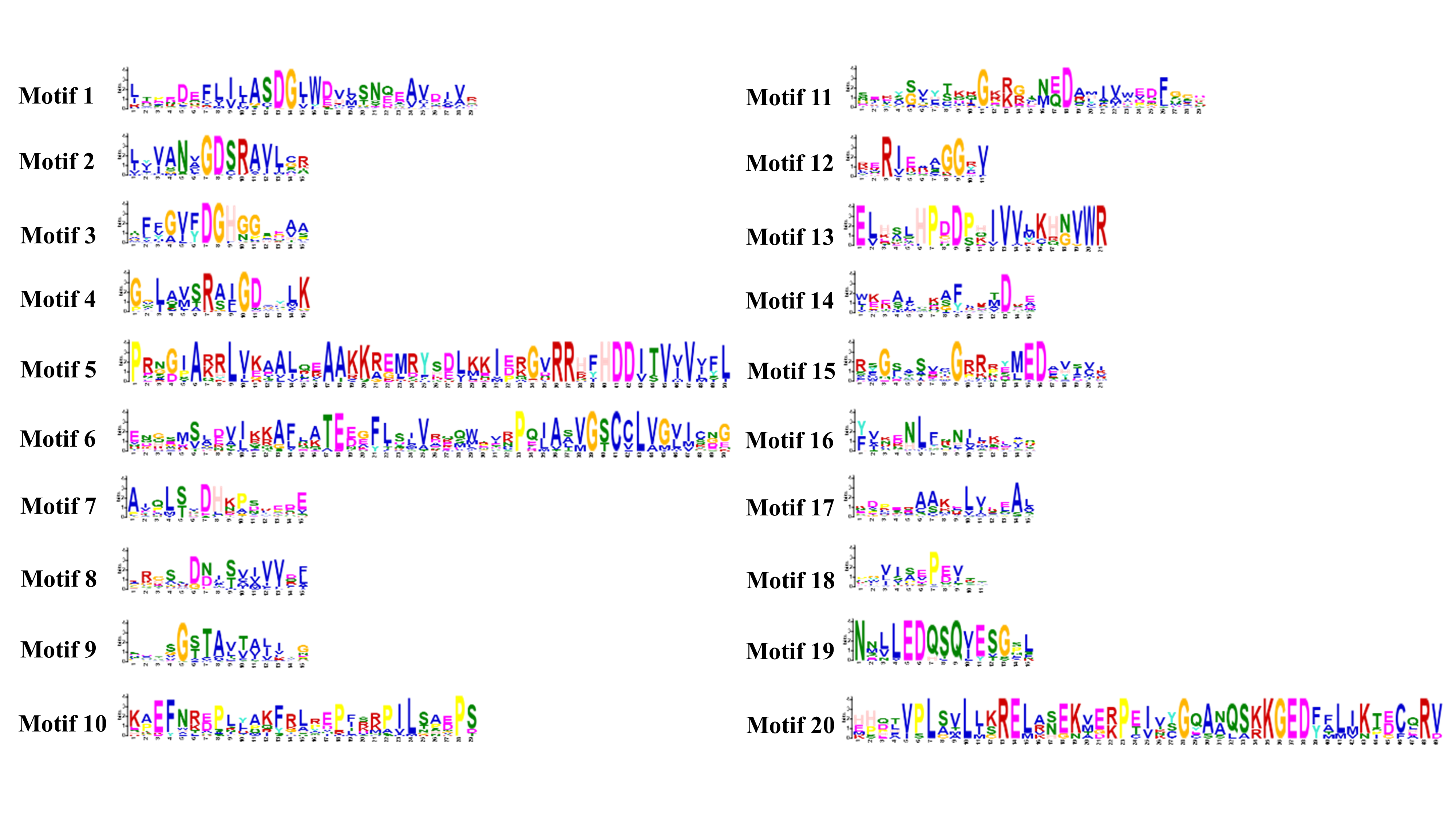

Supplement: S1 Fig — (TIF) [file pone.0298543.s011.tif]

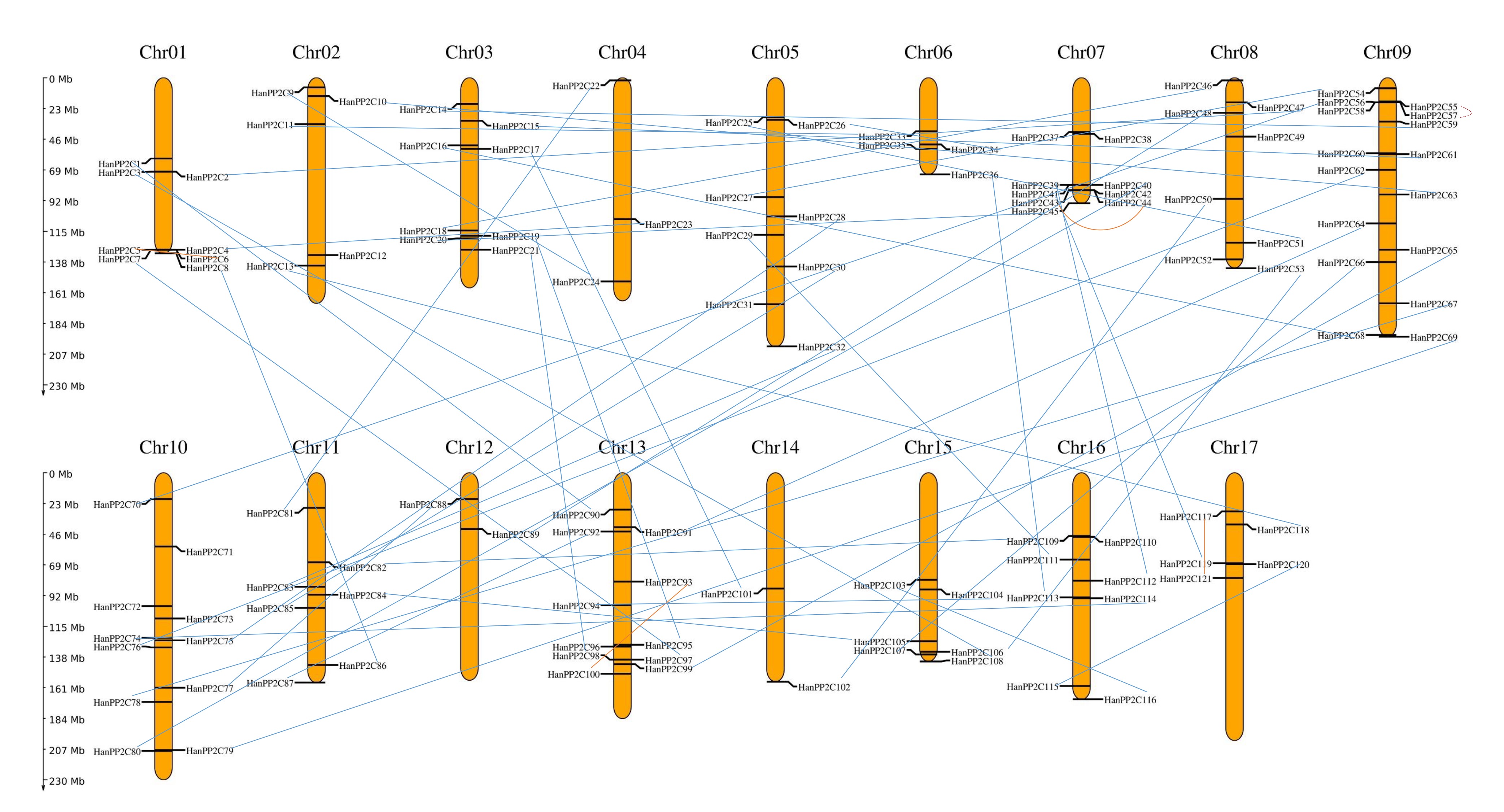

Supplement: S2 Fig — The number of distinct chromosomes is at the top of each chromosome bar. The chromosome-scale is in millions of bases (Mb), indicating the length of each chromosome on the left, using the information retrieved from Phytozome v13. Light orange lines indicate tandem duplications, while light blue lines indicate segmental duplications. (TIF) [file pone.0298543.s012.tif]
